# Ursolic Acid Limits Salt-Induced Oxidative Damage by Interfering With Nitric Oxide Production and Oxidative Defense Machinery in Rice

**DOI:** 10.3389/fpls.2020.00697

**Published:** 2020-06-24

**Authors:** Meijuan Long, Jianyao Shou, Jian Wang, Weizhen Hu, Fakhir Hannan, Theodore M. Mwamba, Muhammad A. Farooq, Weijun Zhou, Faisal Islam

**Affiliations:** ^1^Institute of Crop Science, Zhejiang University, Hangzhou, China; ^2^Zhuji Municipal Agro-Tech Extension Center, Zhuji, China; ^3^Ministry of Agriculture and Rural Affairs Laboratory of Spectroscopy Sensing, Zhejiang University, Hangzhou, China; ^4^Agricultural Experiment Station, Zhejiang University, Hangzhou, China

**Keywords:** NaCl stress, ursolic acid, nitric oxide signaling, sodium, inhibitors, gene expression

## Abstract

Crops frequently encounter abiotic stresses, and salinity is a prime factor that suppresses plant growth and crop productivity, globally. Ursolic acid (UA) is a potential signaling molecule that alters physiology and biochemical processes and activates the defense mechanism in numerous animal models; however, effects of UA in plants under stress conditions and the underlying mechanism of stress alleviation have not been explored yet. This study examined the effects of foliar application of UA (100 μM) to mitigate salt stress in three rice cultivars (HZ, 712, and HAY). A pot experiment was conducted in a climate-controlled greenhouse with different salt stress treatments. The results indicated that exposure to NaCl-induced salinity reduces growth of rice cultivars by damaging chlorophyll pigment and chloroplast, particularly at a higher stress level. Application of UA alleviated adverse effects of salinity by suppressing oxidative stress (H_2_O_2_, O^2–^) and stimulating activities of enzymatic and non-enzymatic antioxidants (APX, CAT, POD, GR, GSH, AsA, proline, glycinebutane), as well as protecting cell membrane integrity (MDA, LOX, EL). Furthermore, UA application brought about a significant increase in the concentration of leaf nitric oxide (NO) by modulating the expression of NR and NOS enzymes. It seems that UA application also influenced Na^+^ efflux and maintained a lower cytosolic Na^+^/K^+^ ratio via concomitant upregulation of *OsSOS1* and *OsHKT1;5* in rice cultivars. The results of pharmacological tests have shown that supply of the NO scavenger (PTI) completely reversed the UA-induced salt tolerance in rice cultivars by quenching endogenous NO and triggering oxidative stress, Na^+^ uptake, and lipid peroxidation. The PTI application with UA and sodium nitroprusside (SNP) also caused growth retardation and a significant increase in Na^+^ uptake and oxidative stress in rice cultivars. This suggests that UA promoted salt tolerance of rice cultivars by triggering NO production and limiting toxic ion and reactive oxygen species (ROS) accumulation. These results revealed that both UA and NO are together required to develop a salt tolerance response in rice.

## Introduction

Salinity is a major ecological constraint that severely affects plant growth and development and decreases crop yield. It has been estimated that one-twentieth of the global total and one-fifth of irrigated lands are salt stressed, consequently leading to US$ 27.5 billion losses of crop yield annually (Qadir et al., 2014). China has approximately 100 million hectares of salt-affected arable land (Yang and Wang, 2015; Islam et al., 2018). The development of salinity in arable land is due to poor irrigation practices, higher rate of surface evaporation, and use of drainage and high-salt-containing groundwater for irrigation, which are key factors causing a 10% increase in saline soil every year, globally (Jamil et al., 2011). Furthermore, due to negative effects of climate-driven changes, rise in global temperature, abrupt seasonal patterns, and anthropogenic activities are exacerbating salt-prone areas, which may result in a loss of 50% arable land at the end of this century (Qadir et al., 2014; Munns and Gilliham, 2015). A soil with an electrical conductivity (EC) of the saturation extract (ECe) of 4 dS/m (approximately 40 mM NaCl at 25°C) and exchangeable sodium of 15% is considered as a saline soil (Etesami and Noori, 2019). Crops grown at high salt concentrations experience cellular ion imbalance, ion toxicity, dehydration, and osmotic stress, which, in turn, results in stomatal closure, reduced carbon supply, and production of reactive oxygen species (ROS). Salt stress tolerance is a complex quantitative trait that is governed by various genetic and non-genetic factors, in which several physiological and molecular mechanisms interact with each other at cellular, organelle, and whole-plant levels, to ensure plant survival and subsequent completion of the life cycle (Hanin et al., 2016; Wu et al., 2019). Crop plants have developed complex strategies to optimize adaptive responses to counter salt toxicity; however, this depends upon the genetic makeup of a particular cultivar/variety and level of salinity in a soil. Many strategies have been proposed to enhance plant salt tolerance, such as exogenous chemical application, breeding, and biotechnological approaches in the past, but commercial successes are limited due to the complex and polygenic nature of salt tolerance mechanisms of crops (Rouphael et al., 2017). In recent decades, intensive research is ongoing on the use of biostimulants/elicitors to ameliorate stress in plants under hostile environmental conditions. It is an attractive alternative way to increase plant performance and productivity (Hayat et al., 2010; Calvo et al., 2014). The exogenous application of these biostimulants/elicitors could balance survival and growth by stimulating diverse arrays of cellular and developmental processes under stress conditions.

Among such biostimulants/elicitors, ursolic acid (UA; 3b-hydroxy-12-urs-12-en-28-oic acid), a natural pentacyclic triterpenoid carboxylic acid, is present in many fruits and vegetables, possessing various biochemical and pharmacological activities like antioxidative, apoptotic, anticancer, and anti-inflammatory properties when used in animal models. For example, it was demonstrated by various studies that UA is a potent free-radical scavenger that restores the level of antioxidant defense-related enzymes (SOD, GR, CAT, GSH, and AsA). It was also reported that UA could inhibit lipid peroxidation by reducing the production of lipid hydroperoxide and thiobarbituric acid-reactive substances in liver microsomes, leukemic cells, and myocardial cell (Wang et al., 2005; Senthil et al., 2007; Kashyap et al., 2016; Yin et al., 2018). It also has the ability to alter the glutathione redox status: GSH:GSSH ratio and could mediate glutathione-dependent antioxidant response. A growing body of evidence suggests that UA acts as a powerful antioxidant, and it scavenges free oxidative radicals, regulates activity of antioxidant enzymes and redox balance, and protects several key enzymes against ROS stress damages (Kashyap et al., 2016; Yin et al., 2018). In addition to this, UA is also used in cancer immunochemotherapy as a biological response modifier because it can elicit NO in a dose-dependent manner via stimulating NOS expression in various animal cell types (Muto et al., 1990; You et al., 2001; Aguirre-Crespo et al., 2006; Ramachandran and Prasad, 2008). These studies suggest that UA is a multi-signaling molecule, can mediate NO production, and also activates immune systems and maintain effective physic-biochemical changes to regulate homeostasis in the organism.

In plants, nitric oxide (NO) elicitors or modulators could play a vital role in plant adaptability to biotic/abiotic stressors. NO is a gaseous signaling molecule that regulates various cellular and molecular processes under normal and stress conditions. In plants, enzymatic biosynthesis of NO is carried out by nitrate reductase (NR) and nitric oxide synthase (NOS), while non-enzymatic generation is carried out via chemical reactions between NOs and plant metabolites (Cooney et al., 1994). Generally, elicitor-induced NO production could strengthen the resistance/tolerance of crop plants under various diverse kinds of environmental stressors. For example, melatonin triggers NO production and reverses oxidative stress by reducing the accumulation of Cd in wheat (Kaya et al., 2019). Elicitor-mediated NO production also inhibited ethylene production to delay leaf senescence in pear. Similarly, elicited NO also restored root growth of Arabidopsis plants via modulation of antioxidant defense (Liu J. et al., 2019; Liu T. et al., 2019). The interplay of NO and elicitor-induced antioxidant defense mitigates adverse effects of lead toxicity in maize plants via ion homeostasis and persevering chlorophyll fluorescence. Recent studies have shown that methyl jasmonate also induced NO production in the soybean cultivars to improve tolerance against cotton leaf worm (Ashry et al., 2018). A growing number of studies are speculating that if interaction of elicitor molecules with each other and with other components of the signaling pathway occurs, their impact on the plant acclimation under stress conditions could be fast and diverse.

In the animal system, UA is widely used as a stress mediator. Despite the pharmacological and clinical importance of UA, little information is available in the literature relating to the effect of UA on the plants under biotic/abiotic stress conditions especially under salinity. Hence, the aims of the current study are (i) to investigate the hazardous physio-biochemical impacts of NaCl on rice cultivars, (ii) to understand how a UA application might reduce such salt induced injurious effects on rice, and (iii) to unveil the underlying mechanism of UA-simulated NO biosynthesis in rice cultivars and how their interaction is involved in salt tolerance response in diverse rice cultivars. This comprehensive examination could improve our understanding of the synchronous signaling pathways involved in the alleviation of salt stress by UA.

## Materials and Methods

### Plant Material and Experimental Design

The healthy and uniform seeds of three rice (*Oryza sativa* L. ssp. *indica*) cultivars, i.e., HZ, HAY, and 712, were obtained from the seed bank of the College of Agriculture and Biotechnology, Zhejiang University, China. These three cultivars were selected because they have been widely grown in the middle and lower reaches of the Yangtze River. The seeds were surfaced sterilized in 0.1% NaClO for 15 min, then rinsed and soaked with distilled water for a further 20 min. The seeds were germinated on moistened filter paper and kept in the darkness for 48 hr, and then in a growth chamber with day/night temperatures of 24/16°C, a 16-h photoperiod, irradiance of 300 μM m^–2^ s^–1^, and relative humidity of 60–70%. The 7-day-old seedlings were transferred to a 96-well rice growth box (13 cm × 9 cm × 12 cm) under half-strength Hoagland solution. After a week of acclimation, a full concentration of Hogland’s solution was supplied to the plants for the rest of the experimental period. Twenty-two-day-old plants were treated with different NaCl salinity (4 dS m^–1^ and 8 dS m^–1^) treatments based upon our previous study (Islam et al., 2016a, 2017). Usually, salinity is measured in units of electrical conductivity (EC), and according to the International Rice Research Institute (IRRI), salinity beyond ECe ∼ 4 decisiemens per meter (dS m^–1^) is considered as moderate salinity while more than 8 dS m^–1^ becomes high for rice plants. The concentration of UA (100) μM was selected based on the preliminary experiment (Supplementary Figure 1). Two days after the pretreatment of ursolic acid (UA 100 μM), plants were exposed to the salinity. The required solution of UA was made each time in dimethyl sulfoxide (DMSO), and then Milli-Q water was added. The leaves of rice stressed plants were sprayed until full wetting, while control plants were sprayed with the same amount of DMSO in water that was used to dissolve UA. Salt stress was applied in a stepwise manner to avoid salt shock by gradually adding NaCl to the nutrient solution with increasing electrical conductivity 2 dS m^–1^ per day. Salt concentration was continually monitored using an electrical conductance meter. Non-stress control plants were maintained in identical nutrient solutions without NaCl addition (Islam et al., 2018). Each treatment was replicated with five biological repeats. The nutrient solution was renewed every 4 days. The experiment was composed of the following treatments:

**Table:** 

**Treatment**	**Description**
Contl	Control with EC 1.2 dS m^–1^
UA	Foliar application of UA 100 μM
S1	4 dS m^–1^ saline treatment
S2	8 dS m^–1^ saline treatment
UAS1	4 dS m^–1^ + foliar application of UA 100 μM
UAS2	8 dS m^–1^ + foliar application of UA 100 μM

The plant leaf samples were harvested after 15 days for morphological and biochemical analyses as described below.

### Second Experiment

Another experiment was carried out under the same conditions to further study the effect of UA on the synthesis of endogenous NO using NO scavenger 2-(4-carboxyphenyl)-4,4,5,5-tetramethylimidazoline-1-oxyl-3-oxide potassium salt (PTI) and NO donor sodium nitroprusside (SNP). Both NO donor and scavenger (100 μM) were sprayed singly on the leaves of plants 2 days before the plants were exposed to the salt stress. The dose selection of PTI and SNP was selected on the basis of previous studies (Esringu et al., 2016; Gupta et al., 2017; da Silva Leite et al., 2019). The 2nd experiment was composed of the following treatments:

**Table:** 

**Treatment**	**Description**
Contl −	Control without saline treatment
Contl +	Control with saline treatment of 8 dS m^–1^
UA −	Ursolic acid (100 μM) + without saline treatment
UA +	Ursolic acid (100 μM) + with saline treatment of 8 dS m^–1^
PTI −	PTI (100 μM) + without saline treatment
PTI +	PTI (100 μM) + with saline treatment of 8 dS m^–1^
SNP −	SNP (100 μM) + without saline treatment
SNP +	SNP (100 μM) + with saline treatment of 8 dS m^–1^
UA + PTI −	Ursolic acid (100 μM) + PTI (100 μM) + without saline treatment
UA + PTI +	Ursolic acid (100 μM) + PTI (100 μM) + with saline treatment of 8 dS m^–1^
SNP + PTI −	SNP (100 μM) + PTI (100 μM) + without saline treatment
SNP + PTI +	SNP (100 μM) + PTI (100 μM) + with saline treatment of 8 dS m^–1^

### Morphological Parameters

The plants were harvested and separated into leaves and roots. The fresh weight of plants was measured immediately after harvesting, while for dry biomass, plants were placed for 5 days in an oven at 80°C (Momoh and Zhou, 2001).

### Chlorophyll Determination

The chlorophyll contents were measured according to the method of Arnon (1949).

### Determination of Malondialdehyde and Reactive Oxygen Species Contents

The malondialdehyde (MDA) concentration was measured according to the method of Zhou and Leul (1998). Hydrogen peroxide (H_2_O_2_) was determined following the procedure of Velikova et al. (2000). Briefly, frozen leaf samples (0.5 g) were homogenized in 0.1% (w/v) trichloroacetic acid (TCA) (5 mL). Absorbance of the leaf samples was read at 390 nm, while the content of H_2_O_2_ was measured by comparing the standard curve of H_2_O_2_. The method of Doke (1983) was followed to determine the superoxide anion (O^2–^) level in leaves of rice cultivars. Lipoxygenase (LOX) activity was determined by observing the increasing absorbance at 234 nm spectrophotometrically, where linoleic acid was used as a substrate (Doderer et al., 1992).

### Biochemical Analysis

Frozen leaf samples (leaves 0.5 g) stored at −80°C were homogenized under liquid nitrogen conditions. The powdered samples were mixed with 50 mM potassium phosphate buffer (pH 7.0) containing 1 mM EDTA Na_2_ and 0.5% PVP (w/v). For ascorbate peroxidase (APX) determination, leaf samples were separately mixed in the abovementioned buffer with the addition of 1 mM ascorbic acid (AsA) in buffer to preserve APX activity. Homogenized samples were centrifuged at 14,000 × *g*: 4°C for 20 min, and the supernatant was stored at −20°C for analysis. Catalase (CAT) activity was determined according to the method of Aebi (1984). A 100-μL enzyme extract was added in a 3-mL reaction mixture. The activity was measured by monitoring the decrease in absorbance at 240 nm. The method of Rao et al. (1996) was used to measure the activity of guaiacol peroxidase (POD). Ascorbate peroxidase (APX) activity was determined by monitoring the change in absorbance at 290 nm for 3 min, followed by the method of Nakano and Asada (1981). SOD activity was estimated according to the method of Dhindsa and Matowe (1981) and expressed by U min^–1^ mg^–1^ protein (one SOD unit was defined as the amount of enzyme needed to produce a 50% inhibition of NBT at 560 nm).

### Determination of Non-enzymatic Antioxidants

The reduced glutathione (GSH) and oxidized glutathione (GSSG) contents were estimated according to the methods of Law et al. (1983) with some modifications as described by Islam et al. (2018). The level of GSH for each sample was obtained by subtracting the GSSG level from the total glutathione, which was expressed as n mol g^–1^ FW. All reagents that were used in GSH/GSSG measurement were prepared in 125 mM NaH_2_PO_4_ buffer, containing 6.3 mM EDTA (pH 7.5).

For assessing proline content, the leaves were homogenized in 3% sulfosalicylic acid and centrifuged at 11,500 × *g*. The supernatant was mixed with acid ninhydrin, glacial acetic acid, and phosphoric acid. After incubating the mixture at 100°C for 1 h and cooling, toluene was added; after several minutes, chromophore containing toluene was read spectrophotometrically at 520 nm (Bates et al., 1973). Assays of glycinebetaine content in rice leaves were performed according to Grieve and Grattan (1983) based on the ability of quaternary ammonium compounds to react with iodine. Dried samples were ground and mechanically shaken with 20 mL of deionized water at 20°C. Extracts were diluted with 2 N H_2_SO_4_ at 1:1 v/v and cooled in ice water for 1 h. Cold KI-I_2_ reagent was added and samples stored at 0–4°C for 16 h and centrifuged at 15,000 × *g* for 15 min at 0°C. The pellet was then dissolved in 1,2-dichloroethane and incubated for 4.5 h, and absorbance was read at 365 nm. A standard curve was established with commercial glycinebetaine.

### Determination of Na^+^ and K^+^

For Na^+^/K^+^ determination, 50–100 mg of dry matter of each sample was subjected to an overnight digestion with HNO_3_/H_2_O_2_ according to the method described by Munns et al. (2010). The content of Na^+^ and K^+^ was determined using atomic absorption spectrometry.

### Analysis of NOS and NR Activities and NO Content

For the measurement of NO content in rice cultivar leaves, 0.5 g sample was homogenized in a buffer and centrifuged at 10,000 × *g* for 30 min at 4°C. The Griess reagent assay was used to determine the NO contents at 540 nm, according to the method of Su et al. (2018). The activity of nitrate reductase (NR) was determined according to the method of Poonnachit and Darnell (2004). The activity of NOS was determined according to the method by monitoring its consumption of NADPH and calculated using the extinction coefficient of NADPH (ε = 6.22 mM^–1^ cm^–1^) (Sun et al., 2014).

### Ultrastructural Observations of Leaves

For transmission electron microscopy (TEM), leaf fragments about 1 mm^2^ without ribs were dipped in phosphate buffer containing 2.5% (v/v) glutaraldehyde for more than 12 h. Later samples were immersed in 1% (m/v) OsO_4_ for 1 h and dehydrated for 15–20 min each with 50, 60, 70, 80, 90, 95, and 100% ethanol series and finally in acetone for 20 min. Finally, specimen was embedded in Spurr’s resin for 12 h and was processed for visualization under TEM (TEM-1230EX, JEOL, Japan).

### Total RNA Extraction, cDNA Synthesis, and qRT-PCR Assay

Total RNA was extracted from leaves of rice cultivars using TaKaRa MiniBEST Plant RNA Extraction Kit (Takara Bio Inc., Japan) according to the manufacturer’s protocol. Spectrophotometry and gel electrophoresis were used to determine the integrity of isolated RNA. PrimerScript^TM^ RT Reagent Kit with gDNA Eraser (Takara Bio Inc., Japan) was used to reverse transcribe 1 μg RNA for qPCR analysis. The iCycler IQ Real-Time Detection System Software was used to calculate the threshold cycle values, and quantification of mRNA levels was calculated according to the method of Livak and Schmittgen (2001). Primers used for the amplification of target cDNAs were designated according to rice genomes available in the databank^1^. The specific primers used for each gene are presented in Supplementary Table S1, and their full-length sequences are available in the databank (see text footnote 1).

### Statistical Analysis

For morphological and physiological parameters, five biological replicates were taken for each treatment × cultivar combination, and three/four plants were bulked together for each replicate. Two-way analysis of variance (ANOVA) was performed to evaluate the significance of variation influenced by cultivars, salt, and UA treatments and their interaction using the SPSS 19.0 version for the first experiment data. Duncan’s multiple range test was performed for multiple comparisons to determine significant differences (*p* < 0.05) between individual testaments. The gene expression data were presented as mean values of four biological replicates (with two technical replicates) with standard error. Three-way analysis of variance (ANOVA) was performed to evaluate the significance of variation for the second experiment.

## Results

### Improvement of Plant Growth and Chlorophyll Content by Pretreatment of UA

Plant fresh and dry biomass parameters were measured to investigate the effect of UA pretreatment on rice cultivars in response to salinity stress. Results showed that salinity decreased the biomass production of all studied rice cultivars compared with control plants. Fresh weight (FW) of rice cultivars HZ and 712 was decreased up to 45% under moderate saline stress treatment (S1), while no significant decrease in FW of these cultivars was observed under higher salinity level (S2). In case of HAY cultivar, the loss of FW was up to 31%; however, no further decrease in FW was measured in plants treated with higher salt stress treatment (S2) (Table 1). Pretreatment of UA (100 μM) significantly alleviated toxic effects of salinity on FW, especially under moderate saline stress (S1) compared with higher saline stress treatment (S2). The cultivar HAY showed the highest degree of shoot FW recovery among all cultivars under both saline stress treatments (S1 and S2). Similar trends of FW losses of root were recorded for all cultivars. UA-mediated alleviation of salinity was most pronounced for cultivar HAY compared with HZ and 712, under higher saline stress treatments (UAS1 and UAS2) (Table 1).

**TABLE 1:** The effect of exogenous application of ursolic acid (UA) on plant biomass production and chlorophyll in the three rice cultivars under saline stress conditions.

**Cultivar**	**Treatment**	**Fresh weight of root (g per plant^–1^)**	**Dry weight of root (g per plant^–1^)**	**Fresh weight of shoot (g per plant^–1^)**	**Dry weight of shoot (g per plant^–1^)**	**Chl *a* (mg g^–1^ FW)**	**Chl *b* (mg g^–1^ FW)**	**T Chl (mg g^–1^ FW)**
HZ	Contl	0.69 ± 0.006a	0.091 ± 0.001a	0.99 ± 0.038a	0.206 ± 0.013a	1.94 ± 0.24a	1.01 ± 0.13ab	2.95 ± 0.33a
	UA	0.63 ± 0.012b	0.049 ± 0.002fg	0.96 ± 0.0020abc	0.159 ± 0.004efg	1.95 ± 0.19a	1.02 ± 0.11a	2.98 ± 0.31a
	S1	0.36 ± 0.014h	0.033 ± 0.001i	0.53 ± 0.084f	0.128 ± 0.003gh	1.40 ± 0.10fgh	0.71 ± 0.04de	2.11 ± 0.18cd
	S2	0.27 ± 0.006i	0.032 ± 0.002i	0.44 ± 0.03f	0.090 ± 0.017cd	0.90 ± 0.08h	0.23 ± 0.01h	1.13 ± 0.18g
	UAS1	0.48 ± 0.017de	0.048 ± 0.005fgh	0.84 ± 0.043ab	0.176 ± 0.005b	1.76 ± 0.18abcd	0.89 ± 0.05bc	2.65 ± 0.31ab
	UAS2	0.41 ± 0.014gh	0.044 ± 0.002gh	0.64 ± 0.078cde	0.126 ± 0.007h	1.30 ± 0.14gh	0.43 ± 0.2g	1.73 ± 0.16def
712	Contl	0.60 ± 0.009b	0.073 ± 0.001bc	1.04 ± 0.064a	0.211 ± 0.009a	1.92 ± 0.19a	1.00 ± 0.06ab	2.92 ± 0.24a
	UA	0.54 ± 0.003c	0.058 ± 0.003de	0.99 ± 0.009bcd	0.142 ± 0.005efg	1.96 ± 0.14e	1.03 ± 0.01a	2.99 ± 0.31a
	S1	0.36 ± 0.029h	0.040 ± 0.02h	0.56 ± 0.066f	0.123 ± 0.006gh	1.63 ± 0.15bcd	0.79 ± 0.043ef	2.42 ± 0.39bc
	S2	0.29 ± 0.040i	0.037 ± 0.001h	0.49 ± 0.049ef	0.157 ± 0.010cd	1.24 ± 0.11g	0.43 ± 0.041g	1.67 ± 0.21ef
	UAS1	0.44 ± 0.023efg	0.046 ± 0.003fgh	0.85 ± 0.115a	0.187 ± 0.009b	1.86 ± 0.19abc	0.89 ± 0.046cd	2.75 ± 0.19ab
	UAS2	0.37 ± 0.026gh	0.050 ± 0.002gh	0.69 ± 0.010def	0.118 ± 0.011h	1.60 ± 0.12cde	0.56 ± 0.034ef	2.16 ± 0.22cdf
HAY	Contl	0.55 ± 0.029c	0.076 ± 0.006b	0.94 ± 0.017abb	0.222 ± 0.009a	1.93 ± 0.19ab	1.02 ± 0.02ab	2.95 ± 0.31a
	UA	0.53 ± 0.006c	0.065 ± 0.007cd	0.98 ± 0.0abc	0.154 ± 0.005e	1.90 ± 0.13ab	1.01 ± 0.01a	2.91 ± 0.27a
	S1	0.46 ± 0.032efg	0.055 ± 0.005ef	0.65 ± 0.061def	0.146 ± 0.006fg	1.53 ± 0.12efg	0.65 ± 0.03ef	2.18 ± 0.22cd
	S2	0.37 ± 0.035h	0.043 ± 0.002ef	0.60 ± 0.095def	0.183 ± 0.010bc	1.23 ± 0.18g	0.35 ± 0.12g	1.58 ± 0.13f
	UAS1	0.51 ± 0.029cd	0.043 ± 0.004ef	0.96 ± 0.058abc	0.177 ± 0.009bc	1.93 ± 0.17ab	0.78 ± 0.04cd	2.71 ± 0.25ab
	UAS2	0.43 ± 0.066fg	0.057 ± 0.010de	0.78 ± 0.075bcd	0.159 ± 0.011cde	1.70 ± 0.16abcd	0.63 ± 0.03ef	2.33 ± 0.29bc
Treatment (T)		**	**	**	**	**	**	**
Cultivar (C)		**	*	*	*	ns	ns	ns
T × C		ns	ns	ns	ns	ns	ns	ns

Data are the means of five replicates ± SE, and three/four plants were bulked for each replicate. Values followed by the different letters indicate significant differences based on two-way ANOVA followed by Duncan’s test (*p* < 0.05) for each cultivar at different saline stress treatments. *p < 0.05; **p < 0.01 level; ns, non-significant. Contl, control; UA-, ursolic acid. S1, 4 dS m^–1^; S2, 8 dS m^–1^; UAS1, Ursolic acid + 4 dS m^–1^; UAS2, Ursolic acid + 8 dS m^–1^.

The dry weight (DW) of shoot was also affected by the salinity. The highest reduction of shoot DW under salinity was noted in cultivar HZ compared with 712 and HAY, respectively. The pretreatment of UA markedly alleviated toxic effects of salinity in cultivars HAY and 712. In case of root DW, more than 60 and 40% of root dry biomass accumulation were decreased in cultivars HZ and 712 under S2 treatment, respectively. The cultivar HAY maintained relatively higher root DW under saline stress conditions (S1 and S2) (Table 1). Pretreatment of UA also mitigated root DW inhibition caused by NaCl more in moderate saline stress conditions (S1) compared with higher saline stress treatment (S2) (Table 1).

The chlorophyll contents such as Chl *a*, *b* and total chlorophyll were significantly decreased under salinity stress treatments (S1 and S2) in a dose-dependent manner, especially in cultivar HZ (highest decrease) (Table 1). However, pretreatment of UA significantly improved pigment production under saline stress conditions and the highest recovery of photosynthetic pigments was noted in cultivar HAY. This suggests that UA application could influence growth under salinity via synthesis of photosynthetic pigments in studied rice cultivars (Table 1).

### UA-Induced Attenuation of Oxidative Stress

**FIGURE 1:**
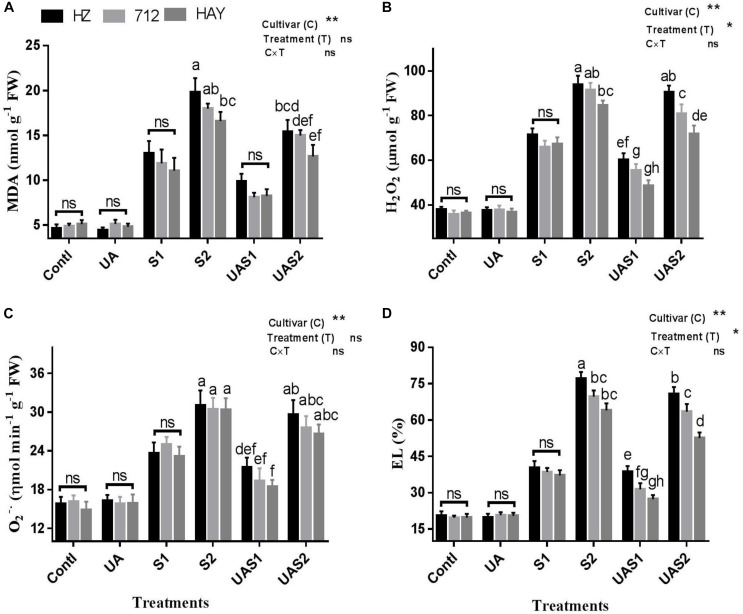
Effect of ursolic acid (UA) on MDA **(A)**, H_2_O_2_ **(B)**, O^2–^ **(C)**, and electrolyte leakage (EL) **(D)** in three rice cultivars (HZ, 712, and HAY) under different salinity treatments. Means with the same letters are not significantly different based on two-way ANOVA followed by Duncan’s test (*p* ≤ 0.05) for each cultivar at different salt stress treatments. The results show the mean ± SE of five replicates, and three/four plants were bulked for each replicate. Error bars represent the standard error. Contl = Control, UA = ursolic acid. S1 = 4 dS m^–1^, S2 = 8 dS m^–1^, UAS1 = ursolic acid + 4 dS m^–1^, UAS2 = ursolic acid + 8 dS m^–1^. *P* ≤ 0.05 (*); *p* ≤ 0.01 (**); ns: not statistically significant (*p* > 0.05).

The extent of oxidative damages caused by NaCl-induced salinity was evaluated by measuring malondialdehyde (MDA), H_2_O_2_, O${}_{2}^{-}$, and electrolyte leakage (EL) (Figure 1). MDA is widely used as an indicator of lipid peroxidation, and electrolyte leakage (EL) is used to assess membrane integrity under stress conditions. MDA was significantly increased in rice cultivars in a dose- and cultivar-dependent manner under salinity treatments (S1 and S2). Approximately, a four- to five-fold increase in MDA was measured in cultivar HZ under moderate (S1) and high saline stress (S2) treatments, respectively (Figure 1). However, pretreatment of UA reduced MDA accumulation significantly in cultivar HAY compared with cultivars 712 and HZ, respectively. Similarly, H_2_O_2_ production was significantly raised by salt stress under higher salt stress treatment (S2) in cultivar HZ, while no significant difference in H_2_O_2_ accumulation was observed among rice cultivars under moderate saline stress conditions (S1), which suggests that H_2_O_2_ production equally affects the physiology of studied rice cultivars (Figure 1). Pretreatment with UA significantly inhibited the NaCl-induced H_2_O_2_ accumulation in rice plants, especially in HAY cultivar. However, less inhibition of H_2_O_2_ in cultivars HZ and 712 compared with HAY cultivar was observed under UAS2 treatment (Figure 1).

The accumulation of superoxide radical (O${}_{2}^{-}$) under saline stress conditions was significantly enhanced as compared with control plants in all cultivars. Among cultivars, the generation rate of superoxide radical was statistically non-significant under saline stress conditions. Pretreatment with UA lowered the generation of superoxide radical considerably, in all rice cultivars compared with their respective salt stress treatments. However, among cultivars UA pretreatment showed a non-significant decrease in superoxide radical production rate (Figure 1).

The electrolyte leakage (EL) of leaves showed that exposure to NaCl significantly increased the EL level with an increase in NaCl concentration in the growth medium. An up to four-fold increase in EL of cultivar HZ and a three-fold increase in 712 and HAY were observed under higher saline stress conditions (S2). Pretreatment of UA significantly inhibited EL in rice cultivar leaves. Among cultivars, the highest EL was measured for cultivar HZ and the lowest for cultivar HAY; this suggests that cultivar HAY maintained superior oxidative and membrane protection characteristics both with and without UA pretreatments (Figure 1).

### UA Mediated Upregulation in Antioxidant Defense Systems

To investigate how UA application influences the antioxidative defense system under salinity, we measured activities of ROS scavenging enzymes, such as SOD, CAT, POD, and APX, in control and salt-stressed plants (Table 2). The exposure to salinity of rice cultivars caused a significant induction in SOD activity. The activity of SOD increased with increasing levels of salinity treatments. The activity of SOD significantly rose up to 2.0- and 3.0-fold under moderate (S1) and higher salinity (S2) levels in cultivars HZ and 712, respectively. The SOD level was increased up to 1.8- to 2.8-fold in cultivar HAY under moderate (S1) and higher salinity (S2) levels (Table 2). However, pretreatment of UA downregulated the activity of SOD in all cultivars under both saline stress treatments. The activities of H_2_O_2_-detoxifying enzymes (POD, CAT, APX, etc.) showed that UA pretreatment accelerated their activities (Table 2). For example, activity of POD was significantly enhanced under saline stress in all cultivars; however, it was sharply elevated in cultivar HAY after pretreatment with UA under moderate and higher saline stress treatments (UAS1 and UAS2). In the case of CAT, the highest rise in its activity under saline stress and UA pretreatment was found in cultivar HAY under all stress treatments (Table 2). The activity of CAT in cultivar 712 was decreased under UAS2 treatment. The activity of APX was not significantly changed in cultivars HZ and 712 under saline stress treatments (S1 and S2) (Table 2). However, cultivar HAY maintained higher levels of APX activity under saline stress treatments (S1 and S2). Pretreatment of UA significantly stimulated APX activity in cultivar HAY under saline stress compared with other cultivars, where APX activity was slightly increased under S1 and S2 treatments (Table 2).

**TABLE 2:** The effect of exogenous application of ursolic acid (UA) on superoxide dismutase (SOD), catalase (CAT), peroxidase (POD), ascorbate peroxidase (APX), glutathione reductase (GR), ascorbic acid (AsA), reduced glutathione (GSH), and oxidized glutathione (GSSG) in the three rice cultivars under saline stress conditions.

**Cultivar**	**Treatment**	**SOD (U mg^–1^ protein)**	**POD (U mg^–1^ protein)**	**CAT (U mg^–1^ protein)**	**APX (U mg^–1^ protein)**	**GSH (nmol g^–1^ FW)**	**GSSG (nmol g^–1^ FW)**	**GR (U mg^–1^ protein)**	**AsA (μmol g^–1^ FW)**
HZ	Contl	25.30 ± 1.51f	13.54 ± 0.76ef	21.86 ± 1.24fg	19.38 ± 1.19h	55.15 ± 2.35g	24.22 ± 2.60f	23.15f ± 1.12g	4.20 ± 0.15g
	UA	30.51 ± 1.82f	14.20 ± 0.59ef	20.75 ± 0.95g	18.73 ± 1.45h	58.72 ± 2.28efg	26.63 ± 1.16def	26.21 ± 1.29efg	4.50 ± 0.25fg
	S1	62.45 ± 2.51c	21.93 ± 1.55cd	25.31 ± 1.12defg	19.47 ± 0.65h	60.37 ± 3.79efg	30.37 ± 1.76cd	23.09 ± 1.56ef	4.97 ± 0.16defg
	S2	83.71 ± 2.03a	25.14 ± 1.52b	25.86 ± 1.21def	20.12 ± 1.19gf	64.60 ± 4.58cde	39.86 ± 1.55a	20.07 ± 1.83g	5.09 ± 0.26defg
	UAS1	50.75 ± 2.47d	31.45 ± 2.25a	36.64 ± 1.72c	24.44 ± 1.56defg	71.25 ± 6.91c	30.74 ± 1.49cd	25.71 ± 2.25efg	7.23 ± 0.9c
	UAS2	71.45 ± 2.34b	30.81 ± 1.55a	41.66 ± 2.07ab	25.42 ± 1.09de	69.97 ± 4.61cd	37.23 ± 1.18ab	28.52 ± 1.25deg	7.40 ± 0.4c
712	Contl	23.26 ± 1.55f	13.67 ± 0.74ef	23.72 ± 1.20efg	18.32 ± 1.08h	56.46 ± 2.52g	23.93 ± 0.94fg	22.33 ± 2.03ef	4.57 ± 0.24fg
	UA	25.98 ± 2.22f	13.80 ± 0.75ef	22.45 ± 1.73fg	20.65 ± 1.52fgh	57.64 ± 4.60efg	24.56 ± 0.59ef	27.39 ± 1.71defg	4.90 ± 0.12defg
	S1	51.68 ± 2.42c	23.42 ± 1.50bc	27.79 ± 0.97de	23.98 ± 1.29defg	63.66 ± 3.89deg	29.82 ± 1.45d	30.06 ± 2.02de	5.20 ± 0.21def
	S2	81.54 ± 2.25a	24.37 ± 0.78b	25.61 ± 1.24defg	21.26 ± 1.80rfgh	69.97 ± 7.39cd	35.94 ± 1.40ab	31.03 ± 1.36cde	5.47 ± 0.15defg
	UAS1	45.34 ± 2.95d	31.95 ± 1.59a	41.31 ± 2.15ab	27.86 ± 1.18bc	89.82 ± 5.63b	27.25 ± 1.51deg	35.73 ± 1.44bc	8.30 ± 0.20c
	UAS2	68.04 ± 4.66b	30.47 ± 1.69a	37.50 ± 2.24bc	29.68 ± 0.69c	93.71 ± 6.24b	34.26 ± 1.65bc	39.63 ± 1.16ab	9.96 ± 1.02b
	Contl	25.19 ± 1.42f	12.71 ± 0.60ef	22.20 ± 1.75fg	20.33 ± 1.50h	55.71 ± 1.45g	22.96 ± 0.94fg	24.64 ± 1.02fgh	4.89 ± 0.24defg
	UA	24.89 ± 1.99f	14.53 ± 1.60ef	24.02 ± 0.62efg	20.17 ± 1.26gh	57.23 ± 4.78fg	23.12 ± 0.72fg	28.71 ± 1.13deg	4.87 ± 0.17efg
HAY	S1	45.36 ± 1.62de	17.48 ± 1.28de	27.45 ± 1.45de	24.74 ± 1.21frh	64.82 ± 3.74cde	28.62 ± 1.34de	30.52 ± 2.58de	5.85 ± 0.28de
	S2	71.29 ± 3.82b	20.08 ± 1.1cd	30.23 ± 1.42d	27.08 ± 1.68bc	68.73 ± 7.81cd	23.20 ± 0.88fg	32.30 ± 2.00cd	6.11 ± 0.28d
	UAS1	40.47 ± 2.55e	23.96 ± 0.97bc	45.32 ± 1.75a	33.87 ± 2.15b	94.78 ± 7.34b	19.86 ± 1.55g	39.53 ± 1.60ab	10.30 ± 0.45b
	UAS2	50.93 ± 3.26d	32.16 ± 0.80a	44.83 ± 1.58a	38.86 ± 1.13a	104.42 ± 9.64a	15.78 ± 1.14h	44.16 ± 2.42a	11.75 ± 0.74a
Treatment (T)		**	**	**	**	**	**	**	**
Cultivar (C)		**	*	**	**	ns	**	ns	*
T × C		ns	*	ns	ns	ns	*	ns	ns

Data are the means of five replicates ± SE, and three/four plants were bulked for each replicate. Values followed by the different letters indicate significant differences based on two-way ANOVA followed by Duncan’s test (*p* < 0.05) for each cultivar at different saline stress treatments. *p < 0.05; **p < 0.01 level; ns, non-significant. Contl, control; UA-, ursolic acid. S1, 4 dS m^–1^; S2, 8 dS m^–1^; UAS1, Ursolic acid + 4 dS m^–1^; UAS2, Ursolic acid + 8 dS m^–1^.

### Effects of UA/Salt on Glutathione–Ascorbate Cycle

The glutathione reductase (GR) and other related enzymes are critically involved in the maintenance of reduced AsA and GSH content in cells to scavenge ROS (Table 2). The salinity treatments (S1 and S2) did not significantly enhance GR activity in the studied rice cultivars. However. GR activity was downregulated (-13%) in cultivar HZ compared with its respective control under higher saline stress treatment (S2). Pretreatment of UA under saline stress significantly enhanced GR activity, especially in cultivars HAY, 712, and HZ, respectively. Generally, GSH concentration was reduced, while GSSG accumulation was enhanced in studied cultivars under saline stress conditions (S1 and S2). However, pretreatment with UA successfully upregulated GSH concentration under saline stress, especially in cultivars HAY and 712, respectively. The concentration of GSSG was elevated in cultivars HZ and 712 after saline stress treatments in a dose-dependent manner. UA pretreatment further declined the GSSG level in cultivar HAY under salinity, while no significant decrease in GSSG level was recorded in cultivar HZ and 712 after UA pretreatment under salinity. Among cultivars, a delicate equilibrium between GSH and GSSG concentration was only observed in cultivar HAY under salinity treatments (S1 and S2). Similarly, salinity also did not significantly influence the content of AsA in rice cultivars. However, pretreatment with UA accelerated the AsA accumulation significantly in all cultivars, but its induction effect was more pronounced in cultivars HAY and 712 compared with HZ, respectively. This suggests that UA strengthened the ROS scavenging system to cope with salt-induced oxidative stress in rice plants.

### UA-Mediated Stimulation of NO Machinery

**FIGURE 2:**
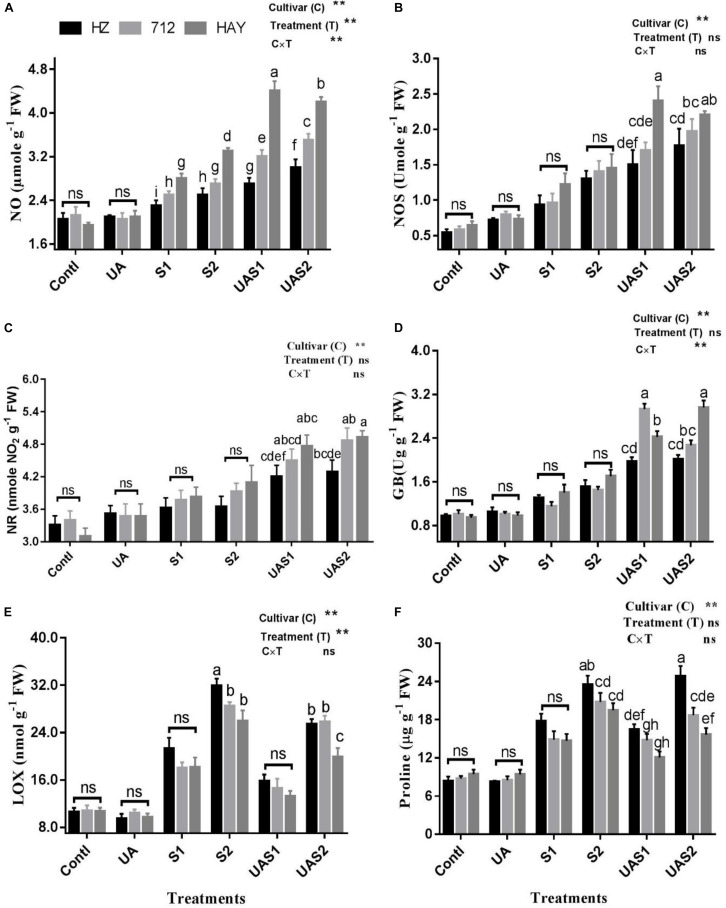
Effect of ursolic acid (UA) on nitric oxide metabolism **(A–C)**, GB **(D)**, LOX **(E)** and proline **(F)** contents in three rice cultivars (HZ, 712, and HAY) under different salinity treatments. Means with the same letters are not significantly different based on two-way ANOVA followed by Duncan’s test (*p* ≤ 0.05) for each cultivar at different salt stress treatments. The results show the mean ± SE of five replicates, and three/four plants were bulked for each replicate. Error bars represent the standard error. Contl = Control, UA = ursolic acid. S1 = 4 dS m^–1^, S2 = 8 dS m^–1^, UAS1 = ursolic acid + 4 dS m^–1^, UAS2 = ursolic acid + 8 dS m^–1^. *p* ≤ 0.05 (*); *p* ≤ 0.01 (**); ns: not statistically significant (*P* > 0.05).

To elucidate the relationship between UA-mediated stimulation of endogenous NO production under salt stress, NO production and its biosynthesis enzyme activities were investigated (Figure 2). Salt stress greatly enhanced NO production in cultivars, while cultivar HZ and 712 showed a non-significant increase in NO production. However, pretreatment of UA under salinity significantly triggered NO production in cultivars HAY, 712, and HZ compared with their respective salt stress treatments, respectively (Figure 2).

NOS activity increased significantly with the enhancement of salt stress levels. The NOS content under moderate salinity treatment increased by 72.7, 64.9, and 90.8% in cultivars HZ, 712, and HAY, respectively (Figure 2). Under higher salinity treatment (S2), NOS levels enhanced by 128.2, 140.8, and 142.3%, respectively, in cultivars HZ, 712, and HAY. At the same time, it is worth noting that the content of NOS continued to increase with the pretreatment of UA, and this increase was particularly significant for cultivar HAY. Under UA and moderate salinity stress treatment (UAS1), NOS content increased up to 96.72%, in cultivar HAY compared with its respective salinity stress treatment (S1) (Figure 2).

Similar to NOS activity, salt stress also increased the content of NR and GB in rice cultivars. The pretreatment of UA under salinity stimulated NR and GB activities compared with their salt respective treatments (S1 and S2). This increase is especially noticeable in cultivar 712 under UAS1 treatment and in HAY under both UAS1 and UAS2 treatments. UAS1 treatment also increased GB content in cultivar 712 by 154.78% compared with S1 treatment. On the other hand, the UAS2 treatment increased GB content by 104.14% compared with S2 treatment in cultivar HAY (Figure 2).

LOX is another important initiator of membrane damage and responsible for lipid peroxidation and formation of superoxide ion. Salinity stress significantly increased the activity of LOX in a dose-dependent manner under moderate and higher saline stress treatments (S1 and S2). Pretreatment of UA under moderate salinity (UAS1) reduced the LOX activity up to 26, 19, and 27% in cultivars HZ, 712, and HAY compared with their corresponding moderate salt stress treatment (S1). Similarly, pretreatment of UA under higher saline stress treatment (UAS2) declined the LOX activity in cultivar HAY compared with other cultivars. This shows that UA pretreatment could lowered the LOX activity to inhibit lipid peroxidation and membrane damage caused by salinity (Figure 2).

Results shown in Figure 2 f revealed an enhancement in proline levels in response to salinity, and the increase was more notable for cultivar HZ under both salt stress treatments (S1 and S2). However, pretreatment of UA under moderate salinity (UAS1) reduced the accumulation of proline non-significantly in all rice cultivars as compared with their respective alone salt stress treatment (S1). Under USA2 treatment, a significant increase in proline accumulation was only observed for cultivar HZ compared with 712 and HAY, respectively. Analysis of data shows that NO production is correlated with the activities of NOS and NR. We observed that endogenous production of NO was not enough in plants of cultivar HZ upon exposure to the salt stress; however, considerable production of NO was observed in cultivar 712 under salinity treatments. However, NO production was significantly raised as the plants of cultivar HAY were exposed to the salinity. It was also observed that pretreatment of UA stimulated the NO production in studied cultivars differently in each cultivar. The highest increase in NO production was observed in cultivar HAY plants compared with cultivars 712 and HZ, respectively. This shows that NO production in cultivars may be a part of the UA-mediated salt stress alleviation mechanism in studied rice cultivars.

### Effect of UA and Salinity on Gene Expression of Antioxidant Defense and NO Metabolism

**FIGURE 3:**
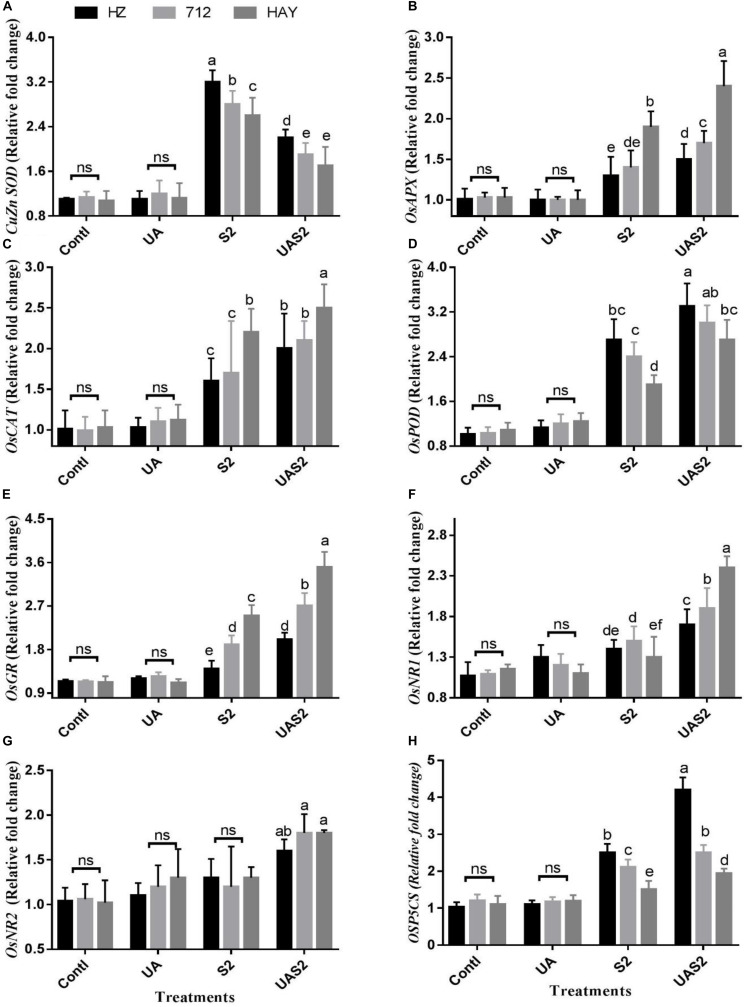
Effect of ursolic acid (UA) on expression of ROS scavenging genes **(A–H)** in leaves of three rice cultivars under controlled and salt stress conditions. Transcript levels were determined by qRT-PCR and calculated by 2^–Δ^ ^Δ^ ^*CT*^ using ubiquitin as an endogenous reference and control plant as a calibrator. Means with the same letters are not significantly different at *p* ≤ 0.05 as determined by Duncan’s test. The results show the mean ± SE of four replicates, and three plants were bulked for each replicate. Contl = Control, UA = ursolic acid. S2 = 8 dS m^–1^, UAS2 = ursolic acid + 8 dS m^–1^.

To better understand UA-mediated alleviation of the oxidative stress mechanism exhibited by the rice cultivars, the transcript levels of antioxidant enzymes were analyzed through quantitative qRT-PCR. CAT transcript abundance under control and UA alone pretreatment did not show any obvious changes. However, exposure to salinity significantly upregulated the expression of CAT in cultivar HAY compared with 712 and HAY. The pretreatment of UA further intensified the expression of CAT in rice cultivars. Similarly, transcription of POD expression was also detected high under saline treatment and UA pretreated salt stressed plants (Figure 3).

The expression levels of cytosolic SOD (CuZn SOD and Mn SOD) were high in cultivar HZ under higher saline stress treatment (S2) compared with other cultivars under said treatment. Pretreatment of UA under higher saline stress (UAS2) treatment reduced the CuZn SOD expression level up to onefold in all cultivars (Figure 3A). However, less suppression of Mn SOD transcript was observed under UAS2 treatment compared with S2 treatment (Supplementary Figure 2), which shows that UA application might reduce the oxidative stress by inhibiting the superoxide anion production in rice cultivars under salinity.

GR and APX transcript accumulation correlated with each other. GR and APX expressions were high in cultivar HAY compared with 712 and HZ under saline stress conditions (S1 and S2), while UA pretreatment under higher saline stress treatment (UAS2) intensified the GR and APX expressions more in cultivar HAY along with others (Figure 3).

NR1 and 2 expression levels did not change significantly under S1 and S2 stress conditions. However, pretreatment of UA under higher salinity (UAS2) only upregulated NR1 expression up to 1.1-fold compared with higher saline stress treatment, in cultivar HAY. However, the expression level of NR1 and 2 was changed up to 0.3- to 0.6-fold for cultivars 712 and HZ under pretreatment with UA and higher saline treatment (UAS2) compared with S2 treatment (Figure 3).

The expression level of proline biosynthesis gene *OsP5CS* was significantly induced under salinity-alone treatments (S1 and S2), and UA pretreatment under salinity (UAS2) further elevated its expression in cultivar HZ. Its expression was increased up to 1.1- and 1.4-fold under S2 and UAS2 treatment compared with control. However, *OsP5CS* expression in cultivar HAY was not significantly changed between S2 and UAS2 treatments (Figure 3).

### Na^+^ and K^+^ Uptake

**FIGURE 4:**
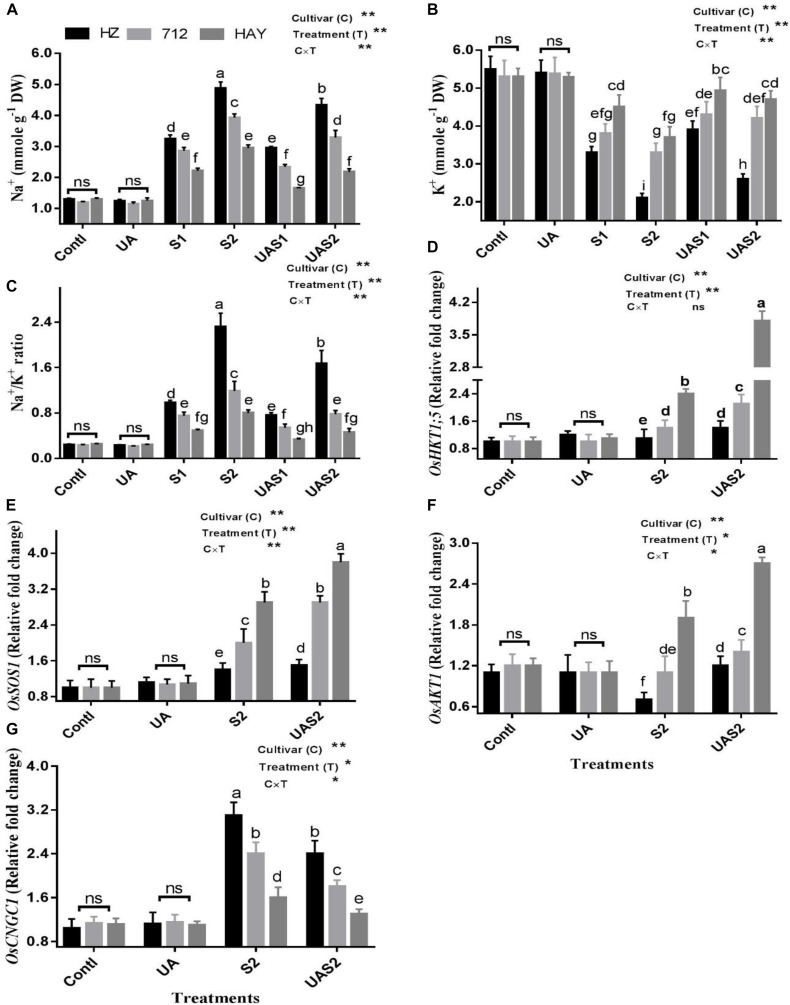
Effect of UA pretreatment on the accumulation of sodium (Na^+^) **(A)**, potassium (K^+^) **(B)**, and Na^+^/K^+^ ratio **(C)** in leaves of three rice cultivars under controlled and salt stress conditions. The results are shown the mean ± SE of five replicates and three/four were plants bulked for each replicate. Error bars represent the standard error. Expression of salt transporter genes **(D–G)** in leaves of three rice cultivars under controlled and salt stress conditions. Transcript levels were determined by qRT-PCR and calculated by 2^–Δ^ ^Δ^ ^*CT*^ using ubiquitin as an endogenous reference and control plant as a calibrator. Means with the same letters are not significantly different based on two-way ANOVA followed by Duncan’s test (*p* ≤ 0.05) for each cultivar at different salt stress treatments. The results are shown the mean ± SE of four replicates and three plants were bulked for each replicate. Contl = control, UA- = ursolic acid. S1 = 4 dS m^–1^, S2 = 8 dS m^–1^, UAS1 = ursolic acid + 4 dS m^–1^, UAS2 = ursolic acid + 8 dS m^–1^. *p* ≤ 0.05 (*); *p* ≤ 0.01 (**); ns: not statistically significant (*p* > 0.05).

As expected, concentrations of Na^+^ and K^+^ were the same in control (Contl) and pretreated UA alone plants (UA). Under moderate saline stress treatment (S1), cultivars HZ and 712 accumulated more Na^+^ compared with cultivar HZ (Figure 4A). Under higher saline stress treatment (S2), 50, 38, and 30% increases in Na^+^ uptake were observed in cultivars HZ, 712, and HAY, respectively. Pretreatment of UA under lower saline stress treatment (UAS1) reduced Na^+^ uptake up to 7, 18, and 25%, while pretreatment of UA under higher saline stress treatment (UAS2) reduced Na^+^ uptake up to 11, 16, and 26% in cultivars HZ, 712, and HAY, compared with their respective salt stress treatments (Figure 4).

In the case of K^+^ retention under salinity, the most significant decrease (40%) in K^+^ concentration was observed for cultivar HZ, while 28 and 15% losses were observed for cultivars 712 and HAY under lower salinity treatment (S1). Under higher salinity treatment (S2), HZ lost a significantly higher amount of K^+^, while HAY maintained a higher retention of K^+^. Pretreatment of UA significantly rescued K^+^ loss, especially for cultivar HAY, which suggests that application of UA strengthens the mechanism that copes Na^+^ entry and K^+^ loss in rice cultivar (Figure 4).

Salt stress significantly increased the Na^+^/K^+^ ratio in all studied rice cultivars. A higher Na^+^/K^+^ ratio was found for cultivar HZ, while a lower ratio was found for cultivar HAY. Pretreatment of UA under salinity treatments (UAS1 and UAS2) significantly reduced the Na^+^/K^+^ ratio for cultivar HAY compared with other cultivars (Figure 4).

### Effect of UA and Salinity on Na^+^ and K^+^ Transporters

An *OsHKT*1;5 is a Na^+^ transporter that maintains efflux of Na^+^ from the xylem sap to prevent its further translocation to the mesophyll cells. The result showed that the expression of *OsHKT1;5* was unchanged in cultivar HZ under S2 treatment, while its expression increased up to 0.4-fold in cultivar 712 compared with its respective control plants under S2 treatment (Figure 4). Pretreatment of UA slightly enhanced the expression of *OsHKT1;5* in cultivar HZ, while the expression level of *OsHKT1;5* was increased up to 0.6-fold under UAS2 treatment. The expression of *OsHKT1;5* was raised up to 2.4 in cultivar HAY under S2 treatment compared with its respective control plants. The pretreatment of UA under higher salinity treatment (UAS2) further enhanced its activity up to 1.6-fold compared with its respective salt stress treatment (S2) (Figure 4).

Salt overly sensitive 1 (*SOS1*) is a Na^+^/H^+^ exchanger that maintains cytosolic ion homeostasis by transporting Na^+^ out of the cell under saline stress conditions. The expression of Os*SOS1* was not changed considerably, in cultivar HZ both with/without UA application under salinity, which suggests that the SOS system was not active in cultivar HZ under salt stress. However, *OsSOS1* transcript abundance was increased up to one-fold under higher salinity treatment (S2) compared with its respective control, in cultivar 712, and pretreatment of UA further accelerated its expression intensity up to 1.5-fold under UAS2 treatment compared with its respective salt stress treatment (S2) (Figure 4). The expression profiling of *OsSOS1* in cultivar HAY under S2 treatment was significantly increased up to 2.9-fold compared with control plants, while under USA2 treatment its activity was further increased up to 3.8-fold compared with control plants (Figure 4).

Inward-rectifying potassium channels, such as *OsAKT1*, mediate K^+^ influx under salinity. The expression level of *OsAKT1* was increased significantly under S2 and UAS2 treatments in cultivar HAY, while a slight increase in *OsAKT1* was observed in cultivar 712 under saline and pretreated UA salt-stressed plants. The expression analysis of *OsAKT1* was downregulated in cultivar HZ under S2 treatment, while UA pretreatment under higher saline stress treatment (UAS2) restored its expression significantly compared with S2 treatment (Figure 4).

Cyclic nucleotide-gated channels (CNGCs) are non-selective cation transporters involved in Na^+^ uptake under salinity. The expression profiling of *OsCNGC1* showed a 3.1- and 2.4-fold increase in its expression in cultivars HZ and 712 under higher saline stress treatment (S2). The pretreatment of UA suppressed its expression up to 23 and 25% in cultivars HZ and 712 under UAS2 treatment. The qPCR expression analysis of *OsCNGC1* showed that its expression was increased up to 44% in cultivar HAY and UA pretreatment reduced its expression up to 19% under UAS2 treatment compared with its respective salt stress treatment (S2). This suggests that pretreatment of UA successively hampers the entry of Na^+^ by suppressing the activity of *OsCNGC1* in rice cultivars especially in cultivar HAY (Figure 4).

### Changes in the Ultrastructure of the Chloroplast and Mitochondria

**FIGURE 5:**
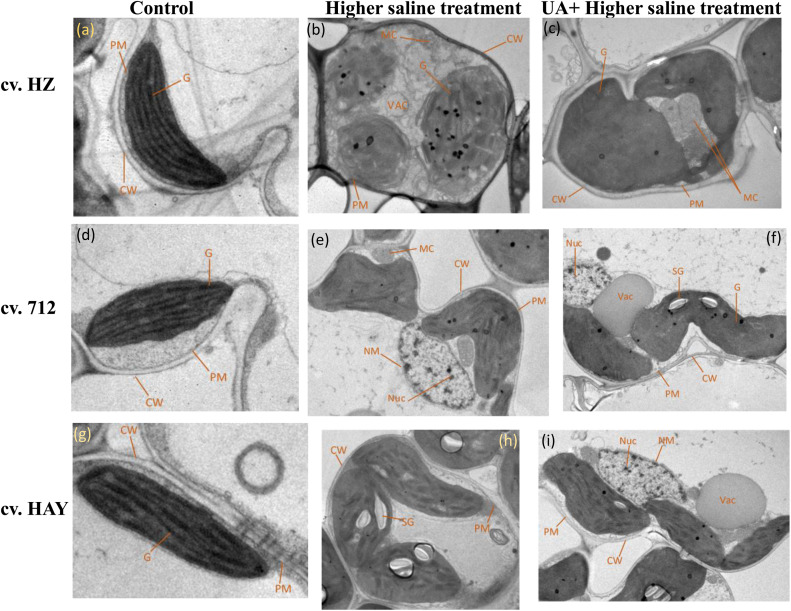
Electron micrographs of leaf mesophyll cells of three rice cultivars (cvs. HZ, 712, and HAY) under Control **(A,D,G)**, S2 = 8 dSm^−1^ **(B,E,H)**, UAS2 = Ursolic acid + 8 dS m^−1^ **(C,F,I)** treatments. PM represents the plasma membrane, G represents the grana, CW represents the cell wall, MC represents the mitochondria, VAC represents the vacuole, NM represents the nuclear membrane, NUC represents the nucleus, and SG represents the starch grain. The experiment was repeated three times with similar results.

Under control conditions, integrated chloroplasts are of typical elongated shape with compactly arranged grana stacks with intact plasma membrane and cell wall (Figures 5A,D,G). Under higher saline stress conditions (S2), the chloroplast envelope was disintegrated and global in shape with reduced grana stacks and distended and loosen thylakoids with mitochondria swelling (Figure 5B) in the plants of cultivar HZ. The chloroplast structure of cultivar 712 under higher saline stress showed swelling of mitochondria with fewer grana stacks. The swelling of mitochondria cristae as well as nuclear membrane was also damaged. The chloroplast structures of cultivar HAY leaves were elongated in shape with fewer stacks of grana. They also possess large starch grains (Figure 5H). The pretreatment of UA under higher saline stress (UAS2) restored the chloroplast structure in cultivar HZ and also minimized the effect of salt stress on the swelling of mitochondria (Figure 5C). Similarly, pretreatment of UA also alleviated toxic effects of salinity on the chloroplast of cultivar 712. The chloroplast was elongated in shape with normal size of mitochondria but with swollen cristae. However, the most pronounced effect of UA alleviation was observed in the chloroplast of cultivar HAY, where UA pretreatment preserved the chloroplast structure, nucleus, and nuclear membrane from toxic effects of salinity (Figure 5I).

### NO Scavenger Impairs the Response of Ursolic Acid–Produced Nitric Oxide in Rice Plants

**FIGURE 6:**
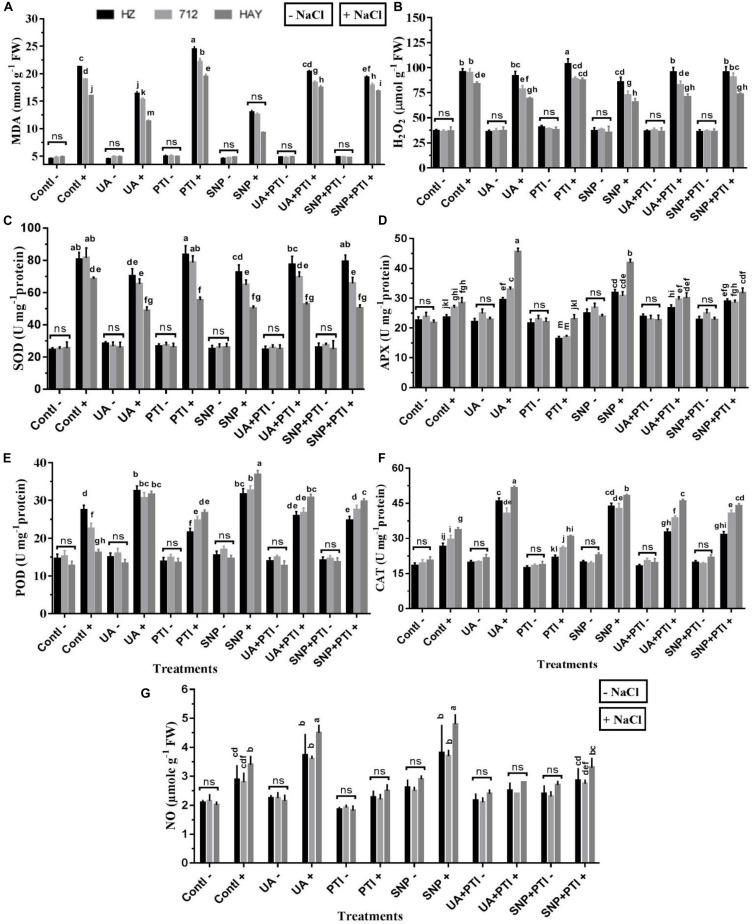
Pharmacological experiment supported the requirement of NO production in Ursolic acid (UA) alleviated NaCl salinity. Redox balance **(A,B)** and antioxidant enzymes activities **(C–F)** were reestablished by UA mediated NO production **(G)** under salinity stress treatment (8 dS m^–1^). Fourteen-day-old rice plants were pretreated with 100 μM UA, 10 μM SNP, 100 μM PTI, alone or their combinations for a day, and then transferred to 8 dS m^–1^ for another seven days. Means with the same letters are not significantly different based on three-way ANOVA followed by Dunca’s test (*P* ≤ 0.05) for each cultivar at different salt stress treatments. The results are shown the mean ± SE of five replicates and three/four plants were bulked for each replicate. Contl = control, UA = ursolic acid, PTI = 2-(4-carboxyphenyl)-4,4,5,5-159 tetramethylimidazoline1-oxyl-3-oxide potassium salt, SNP = sodium nitroprusside. “−” NaCl absent, “ + ” NaCl present, ns: not statistically significant (*p* > 0.05). Three-way interaction of factors is presented in Supplementary Table 2.

To figure out the potential relationship between UA-stimulated production of NO in attenuation of saline stress in rice cultivars, we applied NO scavenger, 2-phenyl-4,4,5,5-tetramethylimidazoline-1-oxyl-3-oxide (PTI), and SNP (sodium nitoprusside), with or without UA-treated rice plants under saline and non-saline stress conditions (Figure 6). We found that UA and SNP alleviated salt stress symptoms, while their toxicity alleviation effects were significantly reversed by the application of PTI on plants of all studied rice cultivars. Application of PTI alone also lowered the NO production under unstressed conditions. Under saline stress conditions, production of NO was significantly suppressed by PTI application compared with salt treatment alone. It was observed that when PTI was mixed with SNP and UA, fluctuations in NO production were clearly observed compared with the SNP and UA alone treatments; however, rice plants performed much better under such treatment regimens (PTI + UA/SNP) compared with plants that were treated only with PTI under salinity treatment. This clearly demonstrates that the NO scavenger disturbs NO metabolism and application of NO donor (SNP) or mediator UA enhances NO production and triggers salt responsive mechanism in treated rice cultivars.

### UA-Mediated NO Is Vital in Reestablishment of Redox Balance in Rice Plants

To understand the underlying molecular mechanism involved in UA-mediated redox modifications, measurement of H_2_O_2_, MDA, and activities of related antioxidant enzymes (SOD, POD, APX, and CAT) was also measured (Figures 6A–F). Results have shown that H_2_O_2_ production, MDA content, and SOD activity were significantly suppressed by UA and SNP application. The activities of ROS scavenging antioxidant enzymes (POD, APX, and CAT) were significantly stimulated by the UA and SNP treatments under saline stress conditions, which indicates that the endogenous production of NO is a key regulator in alleviating salt-induced oxidative stress in studied rice cultivars.

The analysis of antioxidant enzymes under pretreatment of PTI showed that the ROS- scavenging ability of PTI-treated salt-stressed plants was deeply affected and it hampered activities of enzymes (POD, CAT, and APX) compared with SNP and UA pretreatments. The indices related to the ROS production and damage such as H_2_O_2_ and MDA were relatively high in plants treated with PTI under salinity (Figures 6A,B), which clearly favors the assumption of UA-mediated endogenous production of NO in reestablishment of redox balance in saline stress plants.

### UA-Mediated NO Production Influences Salt Transporters and Ion Homeostasis

**FIGURE 7:**
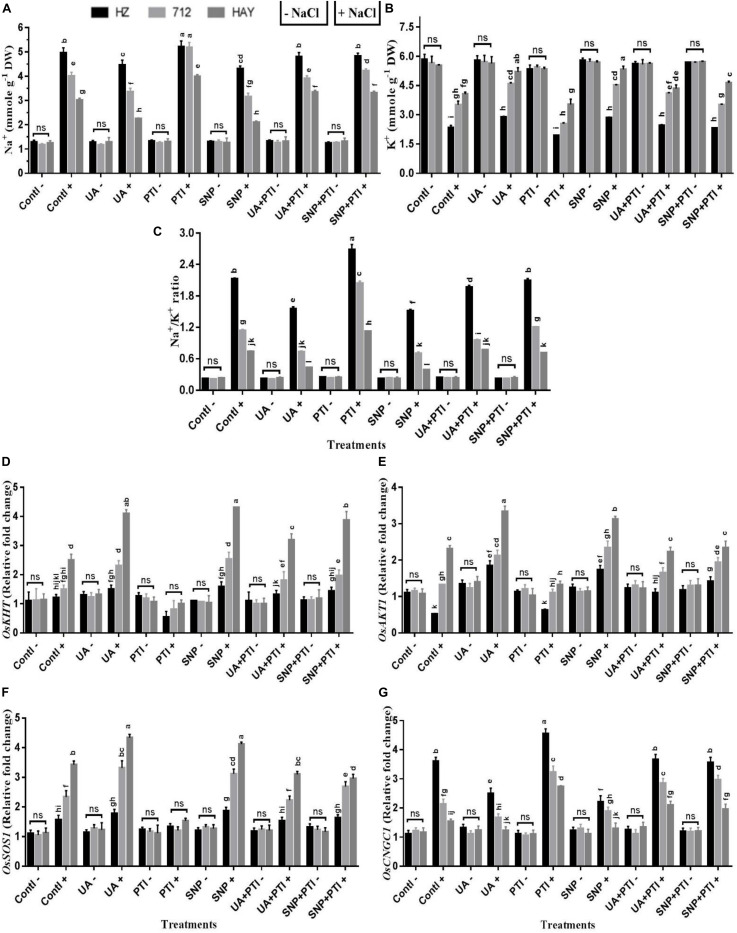
The pharmacological experiments shows that Ursolic acid (UA) modulated ion homeostasis (Na^+^ and K^+^) via NO production in salt stress rice cultivars **(A–C)**. Transcript profiling indicated that activation of Na^+^ and K^+^ transporters and SOS signaling pathway were regulated by UA via NO production in leaves of salt stress cultivars **(D–G)**. Fourteen-day-old rice plants were pretreated with 100 μM UA, 10 μM SNP, 100 μM PTI alone or their combinations for a day, and then transferred to 8 dS m^–1^ for another seven days. Means with the same letters are not significantly different based on three-way ANOVA followed by Duncan’s test (*P* ≤ 0.05) for each cultivar at different salt stress treatments. The results are shown the mean ± SE of five replicates and three/four plants were bulked for each replicate. UA = ursolic acid, PTI = 2-(4-carboxyphenyl)-4,4,5,5-159 tetramethylimidazoline1-oxyl-3-oxide potassium salt, SNP = sodium nitroprusside. “−” NaCl absent, “ + ” NaCl present, ns: not statistically significant (*p* > 0.05). Three-way interaction of factors is presented in Supplementary Table 2.

Ion homeostasis is very crucial for optimum growth of plants. Under salinity, accumulation of toxic ions like Na^+^ and Cl^–^ and loss of K^+^ could inhibit the growth of sensitive crops like rice when exposed to salinity. Therefore, effects of UA and NO, as well as their interplay in ion homeostasis, were also studied (Figure 6). Results showed that the onset of saline stress increased the accumulation of Na^+^, thus leading to a higher Na^+^/K^+^ ratio. By contrast, the pretreatment of UA and SNP declined the Na^+^ uptake and improved K^+^ assimilation, which resulted in a lower Na^+^/K^+^ ratio. However, pretreatment of PTI under salinity reversed the ameliorative effect of UA or SNP compared with saline stress alone treatment (S2) (Figures 7A–C).

To investigate the underlying mechanism of salt tolerance/sensitivity under the influence of different treatment regimens at the transcriptional level, expression profiling of several Na^+^- and K^+^-related genes was analyzed in all studied rice cultivars under UA, NO donor (SNP), and NO scavenger (PTI) pretreatments (Figure 7). As expected, the expression profiling of *OsHKT1;5*, *OsSOS1*, *OsAKT1*, and *OsCNGC1* is consistent with the results of Na^+^ or K^+^ uptake and Na^+^/K^+^ ratio in saline-stressed rice plants. UA and SNP pretreatment stimulated the expression of the *OsHKT1;5*, *OsSOS1*, and *OsAKT* transcript, while a reduction in *OsCNGC1* was observed compared with salinity stress treatment in all rice cultivars (Figure 7). The pretreatment with the NO scavenger (PTI) inhibited the expression of *OsHKT1;5*, *OsSOS1*, and *OsAKT1* transcript, while an increase in expression profiling of *OsCNGC1* was also observed in all rice cultivars under saline stress treatments (Figure 7), which suggests that UA-mediated NO production may reestablish ion homeostasis via modulation of Na^+^ and K^+^ transporters to confer salt tolerance in studied rice cultivars.

## Discussion

The harmful effects of saline stress on plant growth and productivity are well documented. In this aspect, scientists and breeders are employing various conventional and modern approaches to enhance salt stress tolerance/resistance of sensitive crops like rice. Currently, two main annexes, gene editing and development of salt-tolerant germplasms, are widely utilized to obtain salt-tolerant crops. In addition to this, the application of various synthetic/natural bioactive phytochemical metabolites and elicitors is gaining much attention, to ameliorate or enhance plant abiotic/biotic stress tolerance under diverse environmental stressors. In the present investigation, we have employed UA, a pentacyclic triterpenoid, to explore its potential beneficial effects on plant growth under saline stress in three rice cultivars. A wealth of literature provided ample evidence that triterpenoids like UA could modulate multiple targets and signaling pathways in various therapeutics studies in animal models (Shanmugam et al., 2013; Kashyap et al., 2016). However, its beneficial effects on plant biochemical and physiological processes under environmental stress factors are rarely studied.

Exposure of rice cultivars to salt stress significantly hampered plant biomass in a dose- and cultivar-dependent manner. The highest loss in plant biomass accumulation was manifested in cultivars HZ and 712 compared with HAY. This decline in plant biomass accumulation under salinity may be attributed to reduced photosynthetic rate by fractional closure of stomata and reduction in water uptake (Acosta-Motos et al., 2017). However, pretreatment of rice cultivars with UA under salinity considerably prevented a loss in biomass production (Table 1). The decrease in plant biomass may be associated with deterioration of chlorophyll content as, under saline stress, we observed a decrease in Chl *a*, *b* and total chlorophyll of rice cultivars. Previously, it was found that salinity could cause devastation/inhibition of Chl pigment, decrease in total Chl synthesis, and instability of pigment protein complexes (Ahmad et al., 2016). Additionally, salinity also modifies the structure and physiology of organelles involved in photosynthesis such as thylakoid membranes due to ion toxicity and oxidative stress (Figure 1). The stability of chlorophyll molecules under stress conditions depends largely on membrane integrity, which has possibly been maintained by pretreatment of UA. Moreover, UA as an active triterpenoid may enhance the transcript and or translation levels of protein involved in chlorophyll synthesis.

Salt-induced oxidative damage is also determined by measuring MDA, EL, and ROS production, such as H_2_O_2_ and O^2^^.−^ (Figure 1). It is reported that plants experience oxidative stress, when they are exposed to prolonged and lethal salt concentrations. Similarly, a significant increase in H_2_O_2_ and O^2–^ production was observed in rice cultivars under salinity. The increase in ROS is correlated with accumulation of MDA and EL in studied rice cultivars. MDA, a product of lipid peroxidation, has been reported to be a major contributor to cellular damage caused by oxidative stress and is commonly regarded as a marker of oxidative stress. The reduction of MDA and EL was observed when plants were pretreated with UA under salinity, which suggests that UA application reduced ROS production in stressed rice cultivars which resulted in a lower level of lipid peroxidation and EL generation. Previously, in animal models application of UA directly scavenges ROS as a powerful antioxidant and also acts as a signaling molecule (Aguirre-Crespo et al., 2006; Ramachandran and Prasad, 2008; Kashyap et al., 2016). However, the mechanism underlying UA-mediated plant stress responses remains largely unknown.

Plants possess a complex antioxidative defense system comprising non-enzymatic and enzymatic components to scavenge reactive oxygen species (ROS) produced under unfavorable conditions like salinity. Under natural conditions, ROS are generated in a steady-state manner and their appropriate balance is maintained between their syntheses and quenching. Under unfavorable conditions like exposure to salinity, balance of ROS could be disturbed as they gear up rapid production of intra- and inter-cellular ROS levels, which leads to ROS accumulation and subsequent damages to the vital membranes, proteins, and nucleic acids (Sharma et al., 2012). To cope with these conditions, plants trigger ROS scavenging enzyme activities, and their transcript levels (Table 2 and Figure 3). Among them, the first line of defense is SOD, which converts highly reactive OH radical and superoxide (O^·−^) to less toxic H_2_O_2_, which reduces damage to DNA, protein, and membranes (Wang J. et al., 2016; Islam et al., 2017). We found that SOD activity was significantly induced in sensitive cultivars HZ and 712, while HAY cultivar maintained less SOD activity under salinity. Several studies proposed that Mn SOD differed from CuZn SOD in perceiving and scavenging cellular ROS levels (Jebara et al., 2005; Ueda et al., 2013). Overexpression of Mn SOD in transgenic Arabidopsis/rice plants resulted in increased salt tolerance (Wang et al., 2004; Wang F. et al., 2016). Zhao et al., 2018 suggested that Mn SOD compared with CuZn SOD might play a complementary role in the maintenance of total SOD activity and the detoxification of ROS in rice under stress conditions.

Besides this, increased salinity-induced oxidative toxicity in cultivars HZ and 712 may be due to malfunctioning of CAT and APX activities. In plants, CAT is involved in scavenging of hydrogen peroxide with a high turnover rate (Gill and Tuteja, 2010; Chrysargyris et al., 2018; Kataria et al., 2019), and its malfunction could impact ROS detoxification in plant as we observed in cultivars HZ and 712. It is suggested that the plant’s defense system could not effectively compensate for overproduced ROS, if the stress was too harsh. Therefore, reduction in antioxidant enzyme activities may damage plants severely (Haddadi et al., 2016) as we observed for cultivars HZ and 712. However, pretreatment of UA also accelerated the activity of POD in rice cultivars. The POD plays a crucial role in imparting salinity tolerance in rice. The qPCR analysis of antioxidant genes is also correlated with the activities of antioxidant enzymes. Overall, the analysis of antioxidant data reveals that pretreatment of UA protected rice plants from salt-induced toxicity by decreasing oxidative stress and by increasing antioxidant enzyme activities in rice cultivar especially in cultivar HAY.

Ascorbate and GSH are water-soluble, non-enzymatic antioxidants that proficiently scavenge salt-induced ROS and control cellular redox potential (Gill and Tuteja, 2010). The AsA-GSH cycle controls the redox potential of these metabolites, where APX catalyzes the reduction of hydrogen peroxide into water using AsA as electron donor, while GR regenerates AsA with the recycling of GSH. The maintenance of a higher concentration of AsA and GSH/GSSG ratio is vital for protection against salt-induced oxidative stress (Islam et al., 2019). Our results showed that saline stress slightly increased the activities of AsA-GSH cycle enzymes and metabolites; however, this increase was not sufficient to detoxify salt-induced oxidative stress in exposed rice cultivars (Table 2). However, pretreatment of rice cultivars with UA demonstrated the significant increase in APX and GR enzyme activities along with higher maintenance of AsA and GSH pool under salinity (Table 2). Our results are in agreement with many previous studies, where superior maintenance of the AsA-GSH cycle by exogenous application of plant metabolites reduces the salt-induced oxidative stress in exposed plants (Li et al., 2014; Cui et al., 2016; Gupta and Seth, 2020). This suggests the potential of UA in maintaining redox homeostasis to destroy the harmful ROS produced under salinity stress, whereas the role of UA pretreatment in regulating these components has rarely been demonstrated in plants.

One of the vital physiological mechanisms enabling plants to adapt to salinity is synthesis of low-molecular-weight compatible solutes (i.e., proline and GB), to improve water potential and protection of photosynthesis machinery from oxidative damages. It was demonstrated that proline provides protection against NaCl-induced oxidative damage by decreasing the levels of ROS accumulation and lipid peroxidation (Kibria et al., 2017; Kumar et al., 2018). In the present study, a sensitive cultivar HZ had a higher proline content than cultivars 712 and HAY, respectively (Figure 2). These results suggest that cultivar HZ has a proline synthesis mechanism in order to deal with salinity. On the other hand, HAY may have different mechanisms along with proline synthesis to cope with salinity (Akcin and Yalcin, 2016). Similarly, GB also has an important role in the activation of antioxidant enzymes and protection of PSII complex and cell membrane under oxidative stress. In the present investigation, UA pretreatment under salinity accelerated GB accumulation in rice cultivars. These results indicate that exogenous application of UA plays an important role in synthesis of compatible solutes in rice cultivars to maintain water potential and cellular redox balance at optimum levels. Plants experience ion imbalance and metabolic disorder due to excessive accumulation of intracellular toxic ions after the onset of salinity stress. The adverse effects of salt-induced ion toxicity could be ameliorated by reducing uptake or accumulation of Na^+^ or by maintaining a lower cytosolic Na^+^/K^+^ ratio under salt stress (Blumwald, 2000). Plant ability to prevent accumulation of toxic Na^+^ ion in a vital organ like leaf is considered as a key salt tolerance trait. Several authors suggested that accumulation of toxic Na^+^ ion during salinity is correlated to the salinity tolerance in crops (Cuin et al., 2009; Islam et al., 2016a, b). In the present study, salinity stress triggers Na^+^ accumulation in leaves of rice cultivars in a dose-dependent manner. The highest increase in Na^+^ concentration was observed in cultivar HZ followed by 712 and lastly in HAY (Figure 4). Similarly, K^+^ loss was also observed upon exposure to salinity. Maintenance of shoot K^+^ content is important for plants in order to retain growth and development under saline conditions (Islam et al., 2016a, 2017). However, there is no previous report regarding the role of UA on homeostasis of ions. However, NO elicitors and exogenous application of NO prevented the accumulation of Na^+^ in plants via plasma membrane H^+^-ATP-ase activity (Guo et al., 2005; Zhang A. et al., 2007; Zhang F. et al., 2007; Sehar et al., 2019). The present study results showed that pretreatment of UA prevented the excessive uptake of Na^+^ by rice cultivars under salinity treatments; the K^+^ contents were also increased under UA pretreatment when exposed to salinity.

Intracellular Na^+^ is transported out of the cell via a *SOS1* transporter (Shi et al., 2002) or into the root xylem by the high-affinity potassium transporter (*OsHKT1;5*), which is essential for relieving Na^+^ toxicity in stems. The expression level of Os*SOS1* and *OsHKT1;5* was significantly triggered by pretreatment of UA under salinity in cultivar HAY compared with other cultivars (Figure 4). UA pretreatment also meditated the upregulation of *SOS1* expression, which maintains ion homeostasis in cells by transporting Na^+^ out of cells under salinity (Qiu et al., 2002; Peng et al., 2016). Previously, an increase in *OsHKT1;5* activity has been observed in resistant cultivar (Pokkali), whereas its expression was suppressed in sensitive cultivar (FL478) (Platten et al., 2013). We also found that cation transporter *OsCNGC1*, especially transport Na^+^ under salinity conditions (Assaha et al., 2017), may induce Na^+^ accumulation and induce toxicity symptoms in rice cultivars. The suppression of *OsCNGC1* in rice cultivars may be reason of the decrease in uptake of Na^+^ under UA pretreatment in saline stress plants (Senadheera et al., 2009). The superior maintenance of transcript expression of *OsAKT1* in cultivar HAY would explain the increased K^+^ accumulation as compared to cultivars HZ and 712 in UA pretreatment under salinity (Figure 4). We therefore speculated that UA strengthens salt tolerance in rice cultivars partly by modulation in the SOS pathway.

### Potential Mechanism of UA-Mediated Salt Tolerance

Our results show that pretreatment of UA considerably influences the endogenous NO production and its biosynthesis pathway enzymes (Supplementary Figure 3). These findings suggest that UA pretreatment facilitates endogenous NO production, which in turn stimulates post-translational regulatory pathways of NR and NOS. NR exhibits a defensive role in plants by alleviating salt-induced oxidative stress. Induction of NR activity was observed in leaves of salt-treated rice cultivars. Failure to a sharp increase in NR activity under salinity is associated with a reduced supply of NADH, which might be the outcome of disorganization of chloroplasts (Supplementary Figure 3), decline in photosynthesis, respiration, and NADH oxidation (Khator and Shekhawat, 2019). Previous studies demonstrated that many external factors and plant elicitors or phytohormones could trigger NO production under unfavorable environmental conditions. For example, under chilling stress NOS triggers NO-mediated ALA-induced oxidation resistance in leaves of *Elumus* mutants (Fu et al., 2015). Phytohormones like melatonin and jasmonic acid stimulated NOS to produce NO burst (Wang and Wu, 2005). Pretreatment of wheat seedling with glucose activates NR to produce NO under aluminum stress (Sun et al., 2018). It has been reported that endogenous NO production could influence the salinity tolerance of several plants, mostly via stimulation of antioxidant defense mechanisms (Khator et al., 2000; Ali et al., 2017; Gadelha et al., 2017; Klein et al., 2018) and suppression in uptake of toxic ions (Fatma et al., 2016; Gadelha et al., 2017). The UA-mediated endogenous NO production may be partly explained the alleviation of salt stress in rice cultivars in the present study.

To confirm whether the UA is involved in the generation of NO under salinity in studied rice cultivars, we employed several pharmacological experiments using NO donors/or NO inhibitors or scavengers. We found that when an NO scavenger/inhibitor was applied to salt stress plants, severe symptoms of salt toxicity were observed in exposed rice cultivars. The rice cultivars accumulated more Na^+^, and loss of K^+^ was also increased (Figure 7). Similarly, production of ROS (H_2_O_2_, O^2^.^–^) and SOD activity was significantly higher in those plants (Figure 6). A significant decrease in CAT, APX, POD, etc., was also observed in PTI-treated plants, which clearly indicates that NO modulation under salinity could disturb plant responses toward salt tolerance. However, when an NO donor (SNP) or UA was applied to salt-treated plants, a significant increase in NO and NR was recorded. Additionally, enhanced NO production due to UA/SNP pretreatment reduces the Na accumulation, K loss, and ROS burst and strengthens the ROS scavenging enzyme activities. From these pharmacological studies (Figures 6, 7), we could speculate that modulation of NO levels by scavenging NO with PTI, downregulating antioxidant gene expression and activities, and depressing rice cultivar salt tolerance and vice versa.

## Conclusion

This experiment discusses the role of UA to enhance salt tolerance of rice cultivars and the development of seedlings under elevated concentrations of Na^+^ (NaCl-derived salinity). The growth of sensitive cultivar HZ was significantly inhibited under saline stress compared with 712 and resistant cultivar HAY. The physicochemical analysis showed that ROS production, lipid peroxidation, and Na^+^ uptake were enhanced in rice cultivars under saline stress conditions. It was also noted that UA pretreatment enhances endogenous production of NO by enhancing the activities of NR and NOS enzymes. UA-mediated NO production stimulated the expression and activities of antioxidant enzymes like SOD, APX, POD, GR, and CAT in rice plants under salinity treatments, which could explain superior stress tolerance in UA pretreatment rice plants. The results also showed that UA pretreatment suppressed the expression of *OsCNGC1* transcript and triggers the transcript accumulation of *OsSOS1* and *OsHKT1;5* genes to facilitate Na^+^ transport out of the cell. Overall, these findings suggest that UA alleviates salt stress in rice cultivars by reestablishment of redox balance and coordinating NO signal transduction pathways related to the salt stress response at a physiological and transcriptional level. Besides this, it is also possible that salt tolerance of studied rice cultivars under UA application might be improved by acid-medicated pH change in the cells that warrant further investigations and will be explored in our upcoming studies.

## Data Availability Statement

The raw data supporting the conclusions of this article will be made available by the authors, without undue reservation, to any qualified researcher.

## Author Contributions

FI and WZ designed the experiments. ML, JS, WH, FI, FH, and TM performed the experiments and data analyses. FI, MF, JW, and WZ wrote the manuscript.

## Conflict of Interest

The authors declare that the research was conducted in the absence of any commercial or financial relationships that could be construed as a potential conflict of interest.

## Footnotes

1 www.ncbi.nlm.nih.gov/
