# Supplementary material for: Ursolic Acid Limits Salt-Induced Oxidative Damage by Interfering With Nitric Oxide Production and Oxidative Defense Machinery in Rice
Source: Front Plant Sci. 2020 Jun 24;11:697. doi: 10.3389/fpls.2020.00697 (PMC7327119; doi:10.3389/fpls.2020.00697)
Supplement: Supplementary file 1 [file Data_Sheet_1.docx]

**Ursolic acid limits salt-induced oxidative damage by interfering with nitric oxide production and oxidative defense machinery in rice**

Meijuan Long ^1,#^, Jianyao Shou ^2,#^, Jian Wang ^1,3^, Weizhen Hu^4^, Fakhir Hannan ^1^, Theodore M. Mwamba ^1^, Muhammad A. Farooq ^1^, Weijun Zhou^1,3*^, Faisal Islam^1,*^

^1^ Institute of Crop Science, Zhejiang University, Hangzhou 310058, China

^2^ Zhuji Municipal Agro-Tech Extension Center, Zhuji 311800, China

^3^Ministry of Agriculture and Rural Affairs Laboratory of Spectroscopy Sensing, Zhejiang University, Hangzhou 310058, China

^4^Agricultural Experiment Station, Zhejiang University, Hangzhou 310058, China

^*^Corresponding authors. Tel.: +86 571 88982770. E-mail addresses: [wjzhou@zju.edu.cn](mailto:wjzhou@zju.edu.cn); faysal224@yahoo.com

^#^ These authors contributed equally to this work.

**Supplementary Table 1.** Sequences of primer pairs used in real-time PCR.

| **Gene name** | **Gene description** | **Primer sequence** |
| --- | --- | --- |
| ***CAT1*** | Catalase isozyme A | 5- TAAGGCCAGACAATGTCAGATG  3- CAGTGGCATTAATACGCCAGTA |
| ***APX1*** | Cytosolic ascorbate peroxidase 1 | 5- AGTACATTGCCCGTGGTACTCT  3- CGCATTTCATACCAACACATCT |
| ***CuZnSOD1*** | Cytosolic copper/zinc-superoxide dismutase | 5- TGGCAGAGCCGTCGTTGT  3- CGATGATCCCGCAAGCAA |
| ***Mn SOD*** | Manganese superoxide dismutase | 5- TTTGGTTCATTTGAGGCACT  3- GTCCTGGTTAGCAGTTGTTTCC |
| ***POD*** | Peroxidase | 5- TGCTTACCAAGAGCGCTGAA  3- TGATCCAGTCAGAGGCGAGA |
| ***GR1*** | Cytosolic glutathione reductase | 5-TATCCACGGAAAGGAAACC  3-TCCAGGCTGTGGTACTCAC |
| ***NR1*** | Nitrate reductase 1 | 5- AATGAGGTGTGGTTATGGATAG  3-3-AGAAGTAGTGGCCTGGAA |
| ***NR2*** | Nitrate reductase 2 | 5- GAGAGGCCGACCAAGTACGGAA  3-CTGCACGTCTTCGTCTTCACCCTGA |
| ***P5CS*** | Delta-1-pyrroline-5-carboxylate synthase | 5- CAAATGCTCCTTTTAGCCTGTT  3-GCGTTGGTACACAAGTTCTCAG |
| ***OsHKT1;5*** | Sodium transporter Hkt1.5 | 5-CCCATCAACTACAGCGTCCT  3-AGCTGTACCCCGTGCTGA |
| ***OsSOS1*** | Na+/H+ antiporter | 5-ATACTGAGTGGGGTTGTTATTGC  3-AAAGGTAAATTTCAAAAGGTACATGG |
| ***OsAKT1*** | Serine/threonine-protein kinases | 5-GAAACGAGCAATGCGTCAG  3-CTTCTCACACAGCGCTTCC |
| ***OsHAK7*** | Potassium transporter 7 | 5-TGCTGTGACACTTGGTTTCC  3-AAATAACAAGGCGAGCAGGA |
| ***OsCNGC1*** | Non-selective cation channels 1 | 5-TGCAATAGCAAAGCGATACTTG  3-TTTTGGCTTTTGCAACCTCT |
| ***OsUBQ5*** | Ubiquitin 5 | 5-ACCACTTCGACCGCCACTACT  3-ACGCCTAAGCCTGCTGGTT |

**Supplementary Table 2.** Statistical significance (P ≤ 0.05) of the effect of salinity, impact of inhibitors, rice cultivars, and their interaction on various plant parameters with/without UA pretreatment conditions.

| Parameters | Cultivars (C) | | Treatments (T) | | Inhibitors (IN) | C × T | IN × T | C × IN × T |
| --- | --- | --- | --- | --- | --- | --- | --- | --- |
| NO | ** | ** | | ** | | ns | ** | ns |
| MDA | ** | ** | | ** | | ** | ** | ** |
| H2O2 | ** | ** | | ** | | ns | ** | ns |
| SOD | ** | ** | | ** | | ns | ** | ns |
| POD | ns | ** | | ** | | ** | ** | ** |
| CAT | ** | ** | | ** | | ** | ** | ** |
| APX | ** | ** | | ** | | ** | ** | ** |
| Na^+^ | ** | ** | | ** | | ** | ** | ** |
| K^+^ | ** | ** | | ** | | ** | ** | ** |
| Na^+^/K^+^ ratio | ** | ** | | ** | | ** | ** | ** |
| *OsHKT1* | ** | ** | | ** | | ** | ** | ** |
| *OsSOS1* | ** | ** | | ** | | ** | ** | ** |
| *OsAKT1* | ** | ** | | ** | | ** | ** | ** |
| *OsCNGC1* | ** | ** | | ** | | ** | ** | ** |

* = Statistical significance at P ≤ 0.05

** = Statistical significance at P ≤ 0.01

ns = not statistically significant (P > 0.05)

**Supplementary Figure 1.**

**Screening of optimum concentration of UA for plant growth**


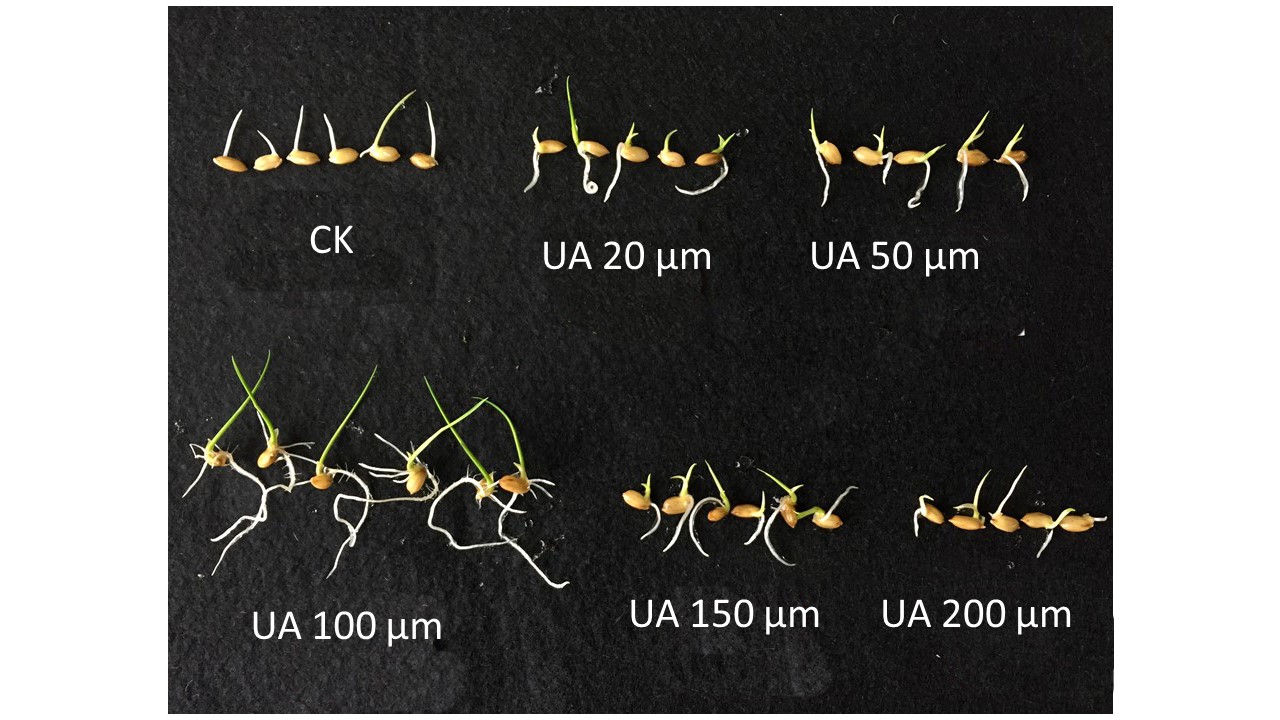


Stock solution of UA was prepared and then we made several working concentrations for the screening test. We have chosen 5 concentrations of UA, i.e 20 µm, 50 µm, 100 µm, 150 µm and 200 µm concentrations. Healthy seed of rice were soaked in 0.1% NaClO for 20 min, then rinsed several times with distilled water and evenly placed in the sterile Petri dish with double filter papers wetted by several concentration of UA solution as described earlier. The incubators were placed at 30 ℃ to accelerate germination under for 48 h. After this time, seed germination was recorded and representative germinated seeds were photographs as shown below.

**Supplementary Figure 1.** Shows the result of preliminary experiment to figure out most effective concentration of UA to carry out further experiment.

**Supplementary Figure 2.**


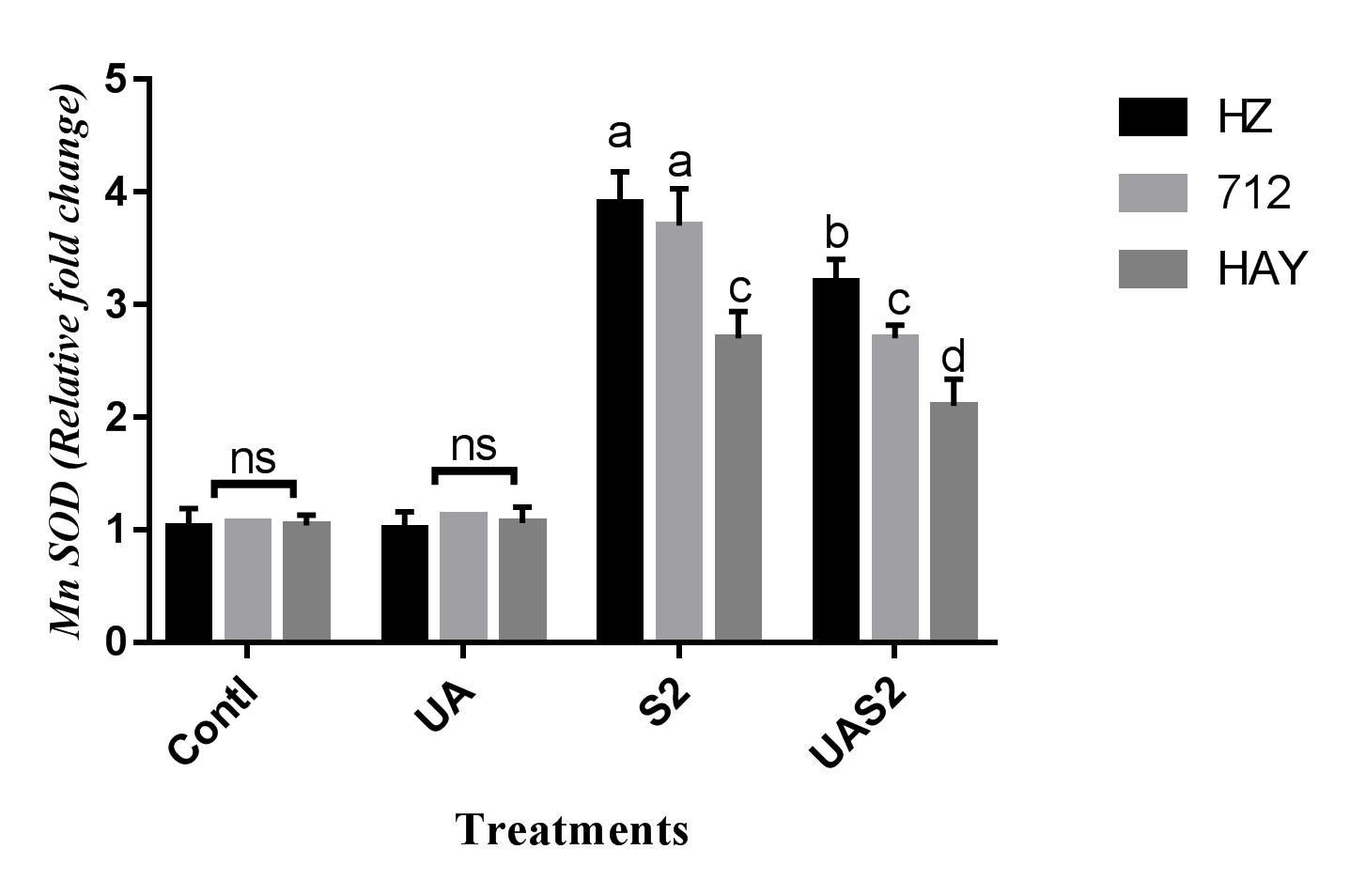


**Supplementary Figure 2:** Expression of *Mn SOD* in leaves of three rice cultivars under controlled and salt stress conditions. Transcript levels were determined by qRT-PCR and calculated by 2^−ΔΔCT^ using ubiquitin as an endogenous reference and control plant as a calibrator. Means with the same letters are not significantly different at P ≤ 0.05 as determined by Duncan’s test. Data represent means ± SD of five replicates. Contl= Control, UA-= Ursolic acid. S2= 8 dSm^-1^, UAS2= Ursolic acid + 8 dS m^-1^.

**Supplementary Figure 3.**


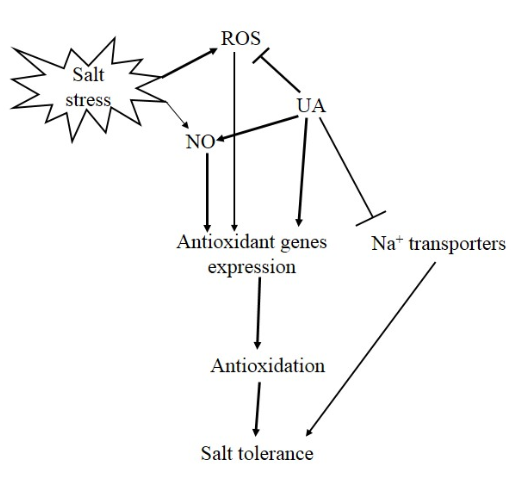


**Supplementary Figure 3:** Proposed model for Ursolic acid (UA) induced involvement in NO mediated oxidative stress tolerance to the salinity in rice cultivars. The bold line represents the strong regulation, and the thin line shows a week regulation.
